# Integrating culture-dependent and culture-independent approaches to explore the diversity of endophytic bacteria associated with *Urtica dioica* L. in Poland with screening of pigment-producing isolates

**DOI:** 10.1186/s12934-026-03077-9

**Published:** 2026-08-04

**Authors:** Natalia Rutkowska, Julia Wojtynek, Weronika Śliżewska, Maciej Nielipiński, Marcin Sypka, Zuzanna Kurek, Oliwia Łukanowska, Weronika Prażanowska, Patrycja Modrzejewska, Bartłomiej Lewocha, Wiktoria Horążak, Igor Naporowski, Weronika Bednarek, Dominika Nielipińska, Piotr Drożdżyński, Olga Marchut-Mikołajczyk

**Affiliations:** 1 Faculty of Biotechnology and Food Sciences, Institute of Molecular and Industrial Biotechnology, Lodz University of Technology, Stefanowskiego 2/22, Lodz, 90-537 Poland; 2 Biotechnology Students Association Ferment, Faculty of Biotechnology and Food Sciences, Lodz University of Technology, Wólczańska 171/173, Lodz, 90-530 Poland

**Keywords:** *Urtica dioica* L., Poland, Bacterial endophytes, Biodiversity, Pigments

## Abstract

**Background:**

Plants can thrive in changing environmental conditions with the help of microorganisms that inhabit their tissues, known as endophytes. However, many endophytes do not grow under standard laboratory conditions, but metagenomic analysis provides insights into microbial diversity and community structure. This study aimed to compare communities of endophytic bacteria from stinging nettle (*Urtica dioica* L.) isolated throughout different regions of Poland, both culturable and non-culturable, and to identify shared bacterial taxa across all locations, which may represent a putative core microbiome.

**Results:**

From all regions, 66 bacterial isolates were obtained. 32% of them were identified, mostly as *Bacillus* and *Pseudomonas* members, which are common endophytes of many plants. Although a substantial proportion of reads remained unclassified, metagenomic data enabled the tentative identification of several genera present across all samples, including *Acinetobacter*, *Bacillus*, *Citrobacter*, *Enterococcus*, *Escherichia*, *Klebsiella*, *Paenibacillus*, *Salmonella*, *Vibrio*, and *Xanthomonas*. Among the isolates, *Exiguobacterium* sp. SP2-R3 exhibited the most intense orange pigmentation. The results indicate that the pigment is putatively quinone-like and demonstrates promising scavenging activity.

**Conclusions:**

The study deepens our understanding of *U. dioica* L. endophytic communities and highlights the value of combining cultivation-based methods with metagenomic approaches. Also, among the isolates with visible potential to produce pigments, the best producer was selected and identified. It is recognized that those isolates may possess additional valuable properties that warrant deeper investigation.

**Supplementary Information:**

The online version contains supplementary material available at https://doi.org/10.1186/s12934-026-03077-9.

## Introduction

*Urtica dioica* L. (common nettle) is a perennial flowering plant in the nettle family (*Urticaceae*), native to temperate climates in Asia, Europe, North Africa, and North America. The plant typically grows in moist environments, such as forests, thickets, roadsides, and marshes. However, it can also often be found in places subject to strong anthropogenic pressure, such as landfills [1]. The flowering period lasts from July to October. Both male and female plant structures have clustered, pendulous inflorescences that are longer than the leaf petiole. The plant is anemophilous, so the flowers themselves are inconspicuous and greenish [2]. The four-stalked stem reaches 30 to 120 cm and, like the leaves. Its equal leaves, which are arranged diagonally, are characteristically ovate with a pointed panicle and serrated edges [3]. A characteristic feature of nettle is the presence of a large number of stinging hairs on the leaves and stems, which, upon contact, cause a reaction known as “hives” attributed to the presence of formic acid and histamine [4–7]. Nettle is rich in organic acids, vitamins such as C, K, and B2, carotenoids, flavonoids, and tannins [8, 9]. The roots contain significant amounts of mucus, waxes, and easily digestible silica [10]. All plant components are used to produce extracts, which have various pharmacological effects due to high levels of flavonoids, tannins, volatile chemicals, fatty acids, steroids, and terpenes, as well as balanced protein content and high levels of minerals and vitamins [6]. Common nettle is primarily used in herbal medicine, where both aboveground and underground parts of the plant are used [3]. It has a diuretic effect, prevents minor gastrointestinal bleeding, and stimulates the secretion of gastric juice. Nettle extract is also used as an additive to shampoos and face masks to regenerate the skin [11].

Endophytic bacteria are microorganisms that live in plant tissues and do not cause any visible negative effects on the plant host due to their presence. Moreover, they support plant growth in both normal and harsh conditions and are thought to be necessary plant partners, also influencing their phenotype [12]. To date, endophytic bacteria have been isolated from various parts of plants, both underground and aboveground components. However, roots harbour a greater abundance of bacterial endophytes in comparison to aboveground structures like stems or leaves [13]. Endophytic bacteria can be divided into groups, reflecting the lifestyle and type of relationship with the plant host. Obligatory endophytes are tightly bound to the host plant and cannot survive independently outside the plant host tissues, while facultative endophytes exhibit a biphasic life cycle, alternating between the plant and its external environment [14].

Bacterial endophytes are of great interest to researchers due to their positive effects on plants, which include growth enhancement, nutrient uptake, and resistance to biotic and abiotic stresses [15]. Endophytes support plant growth through several mechanisms. One of them is the production of phytohormones, organic compounds that can inhibit or stimulate plant growth [16]. They mainly produce indole-3-acetic acid (IAA), gibberellins, ethylene, auxins, and cytokinins [17]. They stimulate the growth of lateral and adventitious roots, increase plant cell division, and promote seed germination [18]. Endophytes also synthesize the enzyme 1-aminocyclopropane-1-carboxylic acid (ACC) deaminase, a protein that is secreted under nitrogen-limited conditions. This enzyme, when exposed to high concentrations of ethylene, breaks down 1-aminocyclopropane-1-carboxylate (ACC) into ammonia and α-ketobutyrate, which can be further recycled by plants [19]. Another enzyme, phosphatase, converts insoluble phosphorus into forms accessible to plants [20]. Furthermore, endophytes can play a crucial role in protecting plants from phytopathogens, not only by producing antimicrobial compounds, but also through direct antagonism or the induction of systemic resistance in the host plant [21]. This group of microorganisms has garnered extensive research attention in recent years, owing to their biomolecules exhibiting a broad spectrum of anticancer, antioxidant, and antimicrobial properties, with potential applications across various industries, including pharmaceuticals, food, and agriculture [22, 23].

A substantial number of endophytes, especially obligatory, do not grow under standard laboratory conditions, presenting a major challenge in studying endophytes associated with certain plant species [14]. Thus, culture-based approaches provide only a partial view of the taxonomic and functional diversity of endophytic communities, as they favour fast-growing and easily culturable microorganisms while excluding a significant portion of the microbiome. Therefore, combining culture-dependent and molecular approaches is necessary to achieve a more comprehensive characterization of endophytic communities. Amplicon-based microbiome profiling, such as 16 S rRNA gene sequencing, provides insights into microbial diversity and community structure directly from plant tissues and helps to overcome the cultivation bottleneck, although it offers limited functional resolution compared to shotgun metagenomics [24, 25].

This study aimed to compare the culturable and non-culturable communities of endophytic bacteria inhabiting common nettle (*Urtica dioica* L.) throughout different regions of Poland. This study is the first research using amplicon-based microbiome profiling of the bacterial microbiome of common nettle in Poland, providing novel preliminary insights into its microbiological diversity influenced by geographical and environmental heterogeneity.

## Materials and methods

### Sample collection

**Fig. 1:**
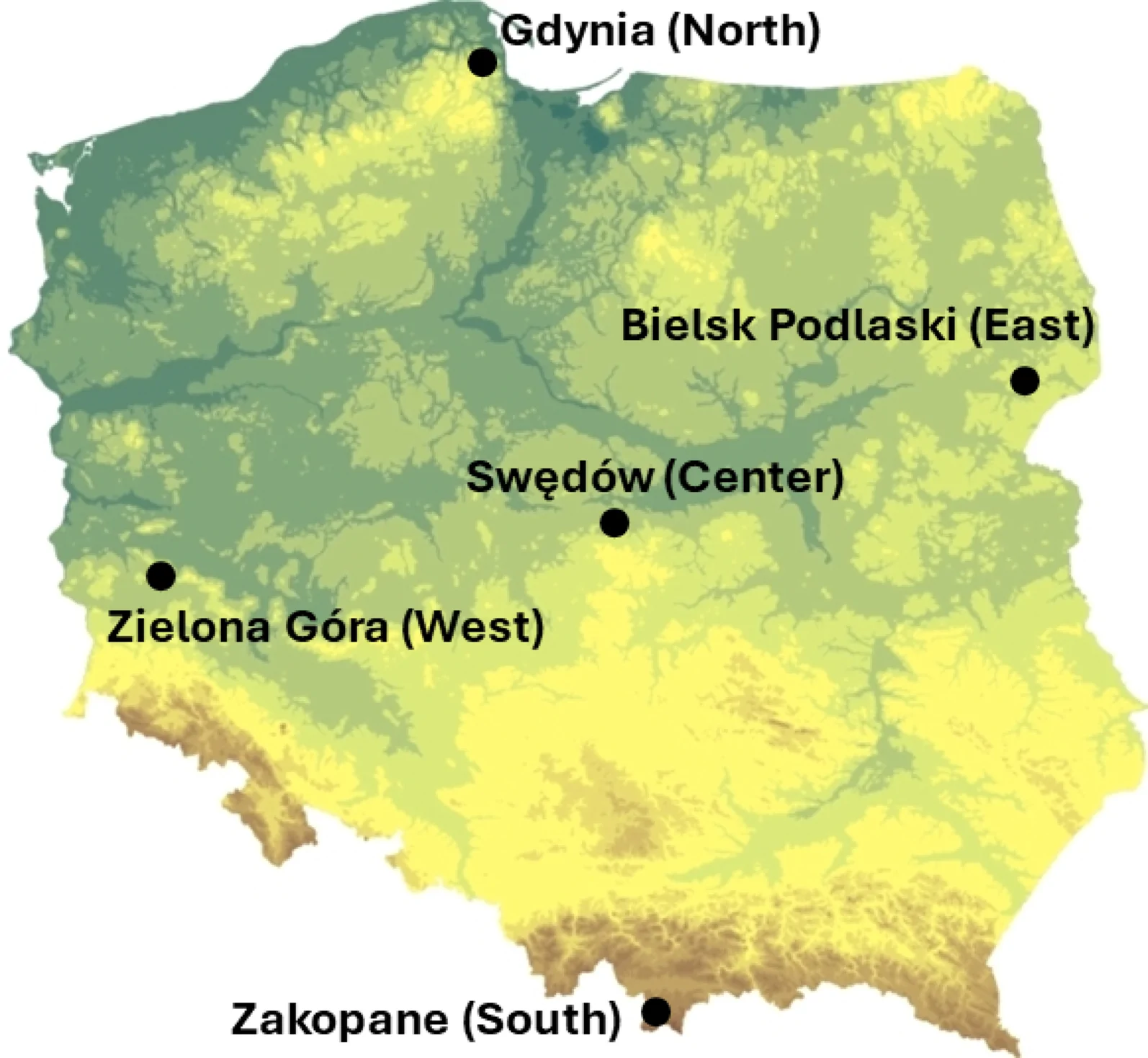
Sampling sites of *Urtica dioica* L. from five different geographic regions in Poland. Base map: “Poland – relief” by Aotearoa (Wikimedia Commons), licensed under CC BY‑SA 3.0. Modified by the authors

Plant material was collected in June and July 2023 from five different regions of Poland: Center, North, East, West and South, according to different geographical regions of Poland (Table 1; Fig. 1).

**Table 1:** Location and characteristics of sampling areas

Location	Region	ID	Coordinates (latitude; longitude) [°]	Characteristics
Gdynia	North	N	54.512584; 18.550718	Shady place in the forest right by the sea, at the foot of the hill
Zielona Góra	West	W	51.9469627; 15.5340544	Forest near the railway tracks
Swędów	Center	C	51.900374; 19.552223	Large nettle field, next to the pond
Bielsk Podlaski	East	E	52.778829; 23.225688	Forest, next to the pond with frogs
Zakopane	South	S	49.298688; 19.963216	Park area

Healthy plants were collected using sterile gloves and a shovel to extract the plant along with the soil. The plants were subsequently placed in a prepared pot, secured and transported to the Institute of Molecular and Industrial Biotechnology at the Lodz University of Technology for further research. On-site, the plants were cleaned with sterile water, left to dry and wrapped in aluminum foil for freezing at -80 °C [26].

### Surface sterilization of the plant material and isolation of endophytic bacteria

Nettles were divided into leaves, stems, and roots; each part was subjected to surface sterilization. Plant parts were soaked in 90% ethanol (3 min), then in 6.25% sodium hypochlorite (5 min) and again in 90% ethanol (30 s) [27]. After soaking five times in sterile deionized water, a small fragment (approx. 1 cm long) was cut from each part. Three fragments of each tissue from each plant were put on Nutrient Agar (NA) plates (peptone, 15 g/L; sodium chloride, 6 g/L; yeast extract, 3 g/L; glucose, 1 g/L; agar, 20 g/L; pH 7.5) and placed in an incubator at 30 °C for 96 h. To ensure the accuracy of the plant sterilization, 200 µl of water after the last wash was also used as control alongside plates with plant samples.

The bacterial colonies grown around the plant tissues were transferred onto richer TSA plates (tryptone, 17 g/L; sodium chloride, 5 g/L; soytone, 3 g/L; glucose, 2.5 g/L; di-potassium hydrogen phosphate, 2.5 g/L; agar; 20 g/L; pH 7.3) and cultivated for another 96 h under the same conditions specified above. Sufficient repetitions were performed to ensure the strain was purified.

### Identification of isolates using MALDI-TOF MS

Isolated strains were cultured on NA plates for 72 h at 30 °C. Single colonies of each isolate were transferred onto a dedicated steel target plate using a sterile inoculation loop in triplicate. Then, 0.5 µL of 25% formic acid (MERCK, Darmstadt, Germany) was added onto biomass, stirred gently, and left until almost dry. Next, 1 µL of saturated matrix solution (40 mg α-cyano-4-hydroxycinnamic acid (α-CHCA; MERCK, Darmstadt, Germany) in 1 mL of acetonitrile/ethanol/water 1:1:1 (v/v) with the final 3% (v/v) trifluoroacetic acid (TFA; MERCK, Darmstadt, Germany) was mixed in, and left to air-dry for 30 min. The samples were analyzed with an AXIMA-iD Plus Confidence MALDI-TOF mass spectrometer (Kratos Analytical Ltd., Manchester, UK and Shimadzu Corporation, Kyoto, Japan). The spectra were collected using Launchpad 2.9 software (Kratos Analytical Ltd., Manchester, UK and Shimadzu Corporation, Kyoto, Japan) in the linear positive ion mode in the mass-to-charge (m/z) ratio range of 2000 to 20,000 Da (50 Hz laser frequency; 90% of laser power). An average mass spectrum (200 profiles; 5 laser shots per profile) was acquired for each sample Minimal intensity of 5 mV, maximal intensity of 2800 mV, minimal *signal-to-noise* (S/N) ratio of 4, peak width of 80 channels, peak smoothing filter width of 50 channels, peak baseline filter of 500 channels and threshold-apex peak detection method of threshold offset 0.040 mV and threshold response of 1.2 x were applied for spectra preprocessing and baseline correction. The overnight cells of *E. coli* DH5α (TAKARA BIO Inc., Kusatsu, Japan) cultured on LB agar (tryptone, 10 g/L; yeast extract, 5 g/L; sodium chloride, 5 g/L; agar, 20 g/L; pH 7.3) plate at 30 °C were used as external calibration. For comparative analysis of the obtained and reference spectra, the SARAMIS Premium 4.11 software (Spectral ARchive and Microbial Identification System; bioMérieux, Craponne, France) was applied. Results were expressed as confidence scores values [%] reflecting similarity of the obtained spectra to reference species-, genus- and family-level spectra present in SARAMIS database. Only scores of ≥ 70% were regarded as sufficient for identification as recommended by the manufacturer.

### DNA extraction and PCR amplification

Genomic DNA from plant tissues was extracted using the GeneMATRIX Soil DNA Purification Kit (EURX^®^, Molecular Biology Products, Gdansk, Poland) following the manufacturer’s instructions. DNA concentration was assessed by A260/A280 using NanoDrop spectrophotometer; its purity and integrity by performing agarose gel electrophoresis. DNA was used as a template for PCR reaction. Gene encoding region V3-V4 of 16 S rRNA was amplified using universal primers (Bakt_341F: CCTACGGNGGCWGCAG and Bakt_805R: GACTACHVGGGTATCTAATCC). The V3-V4 region is widely utilized in microbial community analyses due to its broad coverage of bacterial taxa and reduced amplification of plant-derived plastid sequences [28]. Initial denaturation was carried out at 95℃ for 2 min. The annealing temperature was established at 49 °C, while the elongation was conducted by RUN-HS polymerase (A&A Biotechnology) at 72 °C. Amplification of the genes was carried out in 35 cycles. The composition of reaction mixture was not altered. DNA library preparation and 16 S rDNA amplicon sequencing.

MinION library preparation was performed according to the native barcoding genomic DNA protocol for amplicons (SQK-NBD114.24) with minor modifications. Briefly, the DNA of each sample was repaired and end-prepped, then ligated with barcodes, and, lastly, with adapters. Sequencing was performed on a MinION flow cell (ONT, FLO-MIN114 R10.4.1) connected to a Mk1b device (ONT, MIN-101B) operated by the MinKnow software (ONT 24.06.10). The sequencing run was conducted for 18 h. After sequencing, the raw files in FAST5 format were basecalled (Fast model v4.3.0, 400 bps) using Dorado 7.4.12 with a minimum Q-score of 8 and saved as FASTQ files.

### Metagenomic analysis

Paired-end reads were assigned to samples based on their unique barcodes and truncated by cutting off the barcode and primary sequences, all done in MinKnow software environment. Then, EPI2ME v5.1.14 software was used with the workflow ‘wf-metagenomics’ for the identification of the origin of single reads from amplicon sequencing. Kraken2 2.1.3 was used for the classification of the reads with exclusion of host sequences (database Standard-8), here *Urtica dioica* L.’s chloroplast and mitochondria sequences [29]. α- and β-diversity statistics were calculated using R language in Rstudio interface using mainly *vegan* package [30]. Data for α-diversity calculation was rarefied using *rarefy*(), Shannon and Simpson indices were calculated using *diversity*(), relative abundance was calculated using *decostand*() with ”total” method, distance matrix of samples was calculated using *vegdist*() with Bray-Curtis dissimilarity. Clustering was done using *hclust*() with modified Ward’s method (ward.D2) [31]. Dendrogram was done using *FactoMineR* [32] and *factoextra* [33] packages, heatmap of top found genera was done using *pheatmap* [34] package. Relative abundance data shown on heatmap was scaled rowwise to show relative abundance of genera in the sample; scaling set mean to 0 and standard deviation to 1. Additional packages that were used: *RColorBrewer* was used for color assignment, *ggplot2* [35] for various plot manipulations and *tidyverse* [36] for data manipulation. Venn diagrams were done using Venny v2.1 [37].

### Production and extraction of pigment

Pigment production by the selected bacterial strain was conducted in 500 ml flasks containing TSB medium up to 20% capacity. The pigmented strain was inoculated and incubated at 120 rpm on a rotary shaker, at 30 °C for 72 h. Bacterial biomass was collected by centrifuging at 6500 rpm for 10 min at 4 °C. The obtained cell pellets were then frozen at -20 °C for later use.

Frozen cell pellets (~ 0,5 g) were thawed and mixed with 10 times (w/v) volume of acetone/methanol (7:3, v/v). Extraction was carried out at 60 °C for 2 h at a laboratory shaker (200 rpm) without light access. Bacterial biomass was discarded after centrifugation at 4000 RCF for 10 min at 4 °C [38]. The supernatant was evaporated, and the dry weight of the pigment was measured. The pigment was then resuspended in methanol at a concentration of 10 mg/ml, which was found to be the most effective solvent. Afterwards, the pigment was stored at − 20 °C until further analysis of carotenoids and quinones was conducted.

### Pigment analysis

Pigment was analyzed by ultraviolet (UV)-visible spectroscopy and Fourier transform infrared spectrometry (FTIR). UV-Vis spectra of extracted pigment were measured using a MultiScanGo plate reader (ThermoFisher Scientific, Waltham, MA, USA) in the range from 200 to 700 nm. Organic solvents in which pigments were suspended were used as blank samples.FTIR was analysed on NICOLET 6700 FTIR spectrophotometer (Thermo Scientific) to determine type of the pigment. The spectra were obtained within the wavelength of 4000–500 cm^− 1^ in a transmittance mode.

### Antioxidant activity

The antioxidant activity of the pigment extracted from the bacterial strain SP2-R3 was assessed using both DPPH (2,2-diphenyl-1-picrylhydrazyl) and FRAP (ferric reducing antioxidant power) radical scavenging assays, with ascorbic acid employed as the reference antioxidant. The 10 mg/ml pigment in methanol stock solution was subjected to a two-fold serial dilution, resulting in final concentrations ranging from 10.00 to 0.078125 mg/mL. Solvent used for serial dilutions was methanol in case of DPPH assay, and distilled water for FRAP assay. For each assay, three independent experiments were conducted.

In the DPPH assay [39], 100 µL of the diluted pigment solution was combined with 200 µL of a 0.1 mM DPPH solution prepared in ethanol. The mixture was incubated in the dark for 30 min, after which absorbance was recorded at 517 nm. Methanol was used as a blank. The percentage of radical scavenging activity was calculated using the formula: DPPH inhibition [%] = [(A_blank – A_sample)/A_blank] × 100, where A_blank represents the absorbance of the control (methanol + DPPH) and A_sample is the absorbance of the test solution adjusted for pigment contribution.

For the FRAP assay [40], 10 µL of each sample was added to 180 µL of the freshly prepared FRAP working reagent and incubated at 37 °C in the dark for 15 min. The absorbance was then measured at 593 nm. The FRAP reagent was composed of acetic acid buffer (0.3 M, pH 3.6), 10 mM TPTZ solution, and 20 mM FeCl₃ solution mixed in a 10:1:1 ratio. Water was used both to dilute the samples and as a blank control. A standard curve was constructed using FeSO₄·7 H₂O, and FRAP values were expressed in µmol Fe²⁺/mL based on this calibration.

### Identification of best pigment producer

Endophyte selected as the best pigment producer was grown in LB medium overnight (120 rpm, 30 ℃), and its DNA was extracted by following the manufacturer’s instructions of the GeneMATRIX Bacterial & Yeast Genomic DNA Purification Kit (EURX^®^, Molecular Biology Products, Gdansk, Poland). Its concentration and purity were checked before PCR. PCR was run as described above. Sequencing of the 16 S rDNA was outsourced. Obtained consensus sequence from Sanger sequencing was compared to those in the NCBI database using blastn tool [41]. A phylogenetic tree was constructed with MEGA11 software [42] based on the 16 S rDNA sequences of the strains closest to isolate, using Neighbor-joining method with 1000 repeats of bootstrap [43].

## Results and discussion

### Isolation and selection of bacterial endophytes

**Fig. 2:**
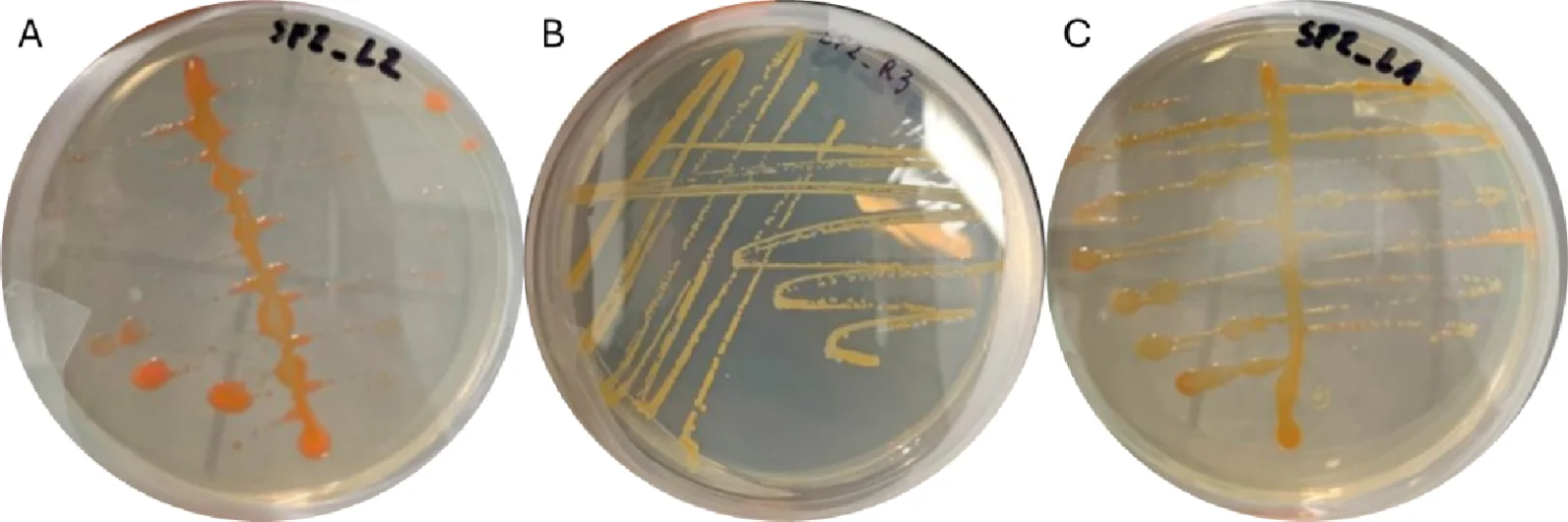
Examples of the strains obtained. **A** SP2_L2; **B** LP2_R3; **C** SP2_L1;

Plant samples were collected at five locations, corresponding to the different geographic regions of Poland, during late June and early July. The surface-sterilized fragments of leaves, stems, and roots were placed on petri dishes with NB media. Following the incubation time, plates with visible bacterial colonies growth were collected. All morphologically distinct colonies from each plate were inoculated separately on NB agar plates. After incubation, 66 pure bacterial isolates were obtained. Figure 2 shows sample plates with isolated organisms.

**Fig. 3:**
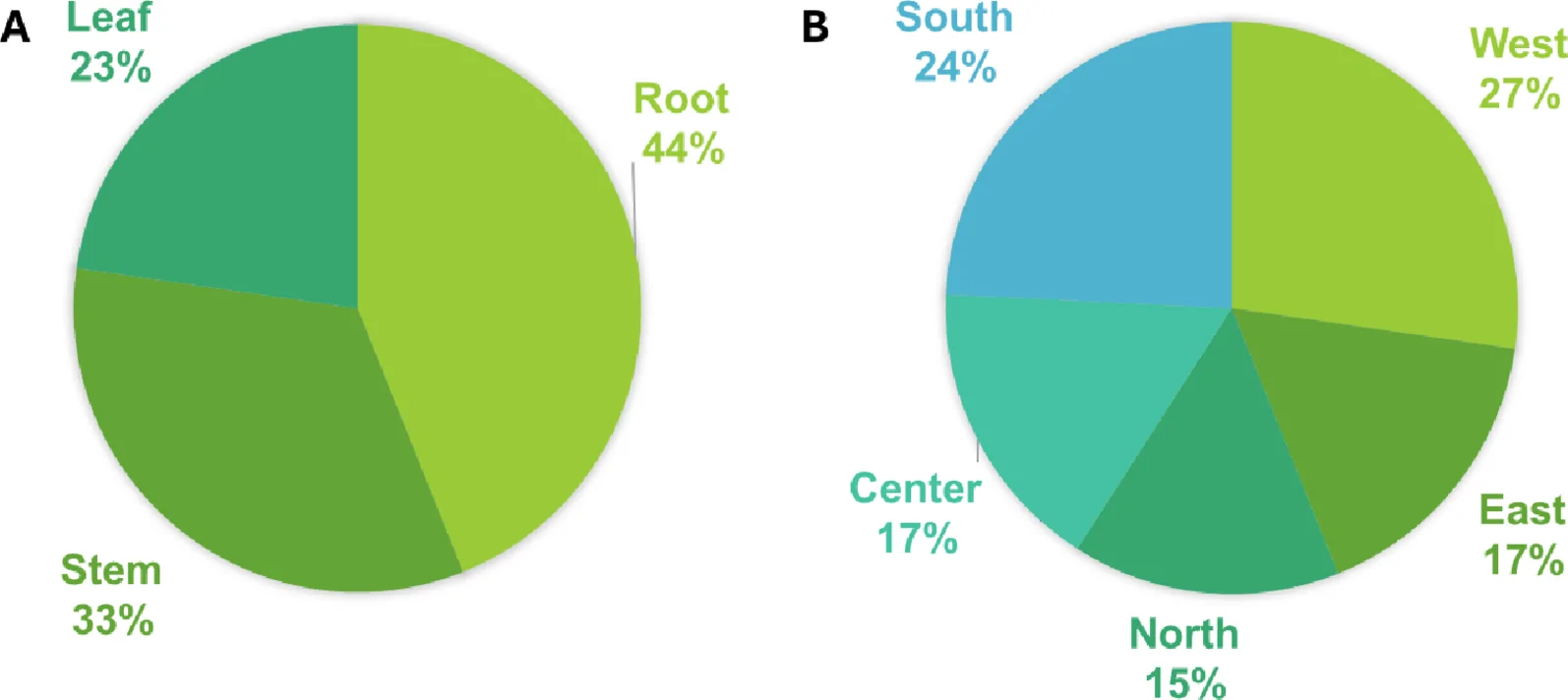
Abundance of isolates within **A** tissues, **B** isolation regions

The abundance of obtained culturable strains varied depending on tissue and isolation region (Fig. 3). About 44% of the isolates originated from roots, 33% from stems, and 23% from leaves. Since most endophytes originate from soil, which is rich in microorganisms, and only some of them are mobile and able to transfer to higher parts of plants, this observation is not surprising [17].

In our earlier study on bacterial endophytes associated with *Urtica dioica* L., culturable isolates obtained from the roots showed the highest abundance from all tissues [26]. Similarly, Passari et al. (2015) reported bacterial endophytes from seven different medical plants, noting distribution levels of 52.3% in roots, 21.4% in stems, and 14.2% in leaves [44]. Abdelshafy Mohamad et al. (2020) observed a comparable pattern in *Thymus vulgaris*, where root-derived isolates accounted for 41.9%, followed by stems at 32.5% and leaves at 25.5% [45]. In some cases, a notable dominance in roots is observed, exampled by *Arctium lappa* L., where root isolates constituted 77.1% of all [46]. A clear trend indicates a greater abundance of bacterial endophytes in roots as compared to stems and leaves is clearly observed among bacterial endophytes from medicinal plants. Even though endophytes can enter plants via stomata and hydathodes in the epidermis, most stem and leaves of residents have been spread from roots to upper parts of plant. This means that they can be transferred to new plants through seeds [47, 48].

### MALDI-TOF MS identification

**Fig. 4:**
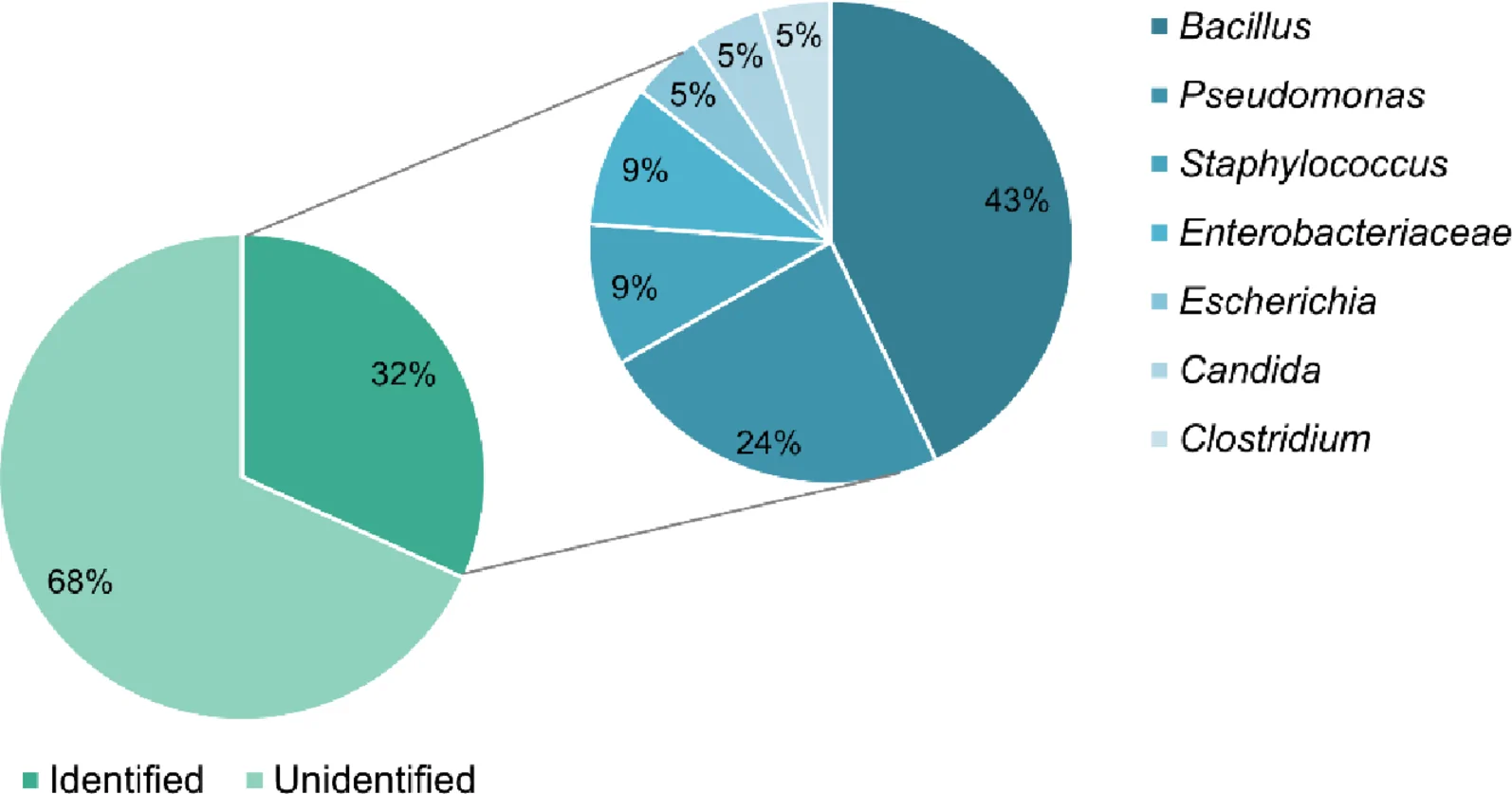
Results obtained from MALDI-TOF MS analyses

Preliminary taxonomic identification of bacterial isolates using the MALDI-TOF MS technique was performed for all strains isolated from samples of roots, leaves, and stems of nettle collected from 5 regions in Poland that could grow in laboratory conditions. The MALDI-TOF MS analysis allowed the putative identification of the 21 strains, which constitute 31.82% of all samples (Fig. 4). The details of the sample results were shown in Table 2. As a result, representatives of *Bacillus* and *Pseudomonas* were the most abundant among all identified strains. The most common endophytic bacteria among identified strains belonged to the genus *Bacillus (B. cereus*,* B. megaterium*,* B. coagulans*,* B. amyloliquefaciens)* and *Pseudomonas*, especially *P. fluorescens* and *P. putida.* Three bacterial isolates were assigned to the *Enterobacteriaceae* family. Such results are not surprising, as those genera are present abundantly in the environment, including soil and water, and are linked to various ecological functions [49, 50]. The identified strains were mostly obtained from the stem and root, at 42.86% and 38.10%, respectively, while those obtained from leaves accounted for only 19.05%.

**Table 2:** Results of identification using MALDI-TOF MS

Isolate	Region	Tissue	Identification	% of identity*
WP1_L1	West	Leaf	*Bacillus weihenstephanensis*	84.0 ± 1.0
WP1_S1		Stem	*Pseudomonas putida*	76.0 ± 0.2
WP1_S2		Stem	*Pseudomonas putida*	82.9 ± 1.4
WP2_R3		Root	*Bacillus megaterium/coagulans/amyloliquefaciens*	84.0 ± 3.2
WP2_S1		Stem	*Bacillus coagulans/megaterium*	82.6 ± 1.3
SP1_S3	South	Stem	*Bacillus cereus* group	96.9 ± 6.6
SP1_R1		Root	*Staphylococcus aureus*	81.2 ± 5.6
SP1_R3		Root	*Enterobacteriaceae* family	87.0 ± 3.6
SP1_R5		Root	*Enterobacteriaceae* family	84.1 ± 4.4
SP2_L2		Leaf	*Escherichia coli*	94.7 ± 0.1
SP2_R1		Root	*Pseudomonas fluorescens*	94.5 ± 0.0
EP1_R2	East	Root	*Pseudomonas fluorescens*	94.5 ± 0.3
EP1_S4		Stem	*Bacillus cereus* group	94.5 ± 2.2
EP1_L1		Leaf	*Candida krusei*	78.5 ± 6.0
EP2_R3		Root	*Bacillus cereus* group	91.8 ± 0.5
CP1_S1	Center	Stem	*Bacillus weihenstephanensis*	76.8 ± 4.2
CP1_S2		Stem	*Bacillus cereus* group	96.9 ± 0.9
CP1_S3		Stem	*Staphylococcus epidermidis*	85.4 ± 5.3
NP1_L3	North	Leaf	*Bacillus weihenstephanensis*	99.9 ± 0.0
NP1_S1		Stem	*Clostridium botulinum*	77.5 ± 6.7
NP2_R4		Root	*Pseudomonas fluorescens*	85.5 ± 4.8

* average of three replicates ± standard deviation

Some of the identified isolates were found to belong to species of well-known opportunistic pathogens such as *Staphylococcus aureus*, *Staphylococcus epidermidis*, and *Clostridium botulinum* [51]. Additionally, one of the tested strains was identified as the yeast *Candida krusei* (78.5% confidence), which is also recognized as a human pathogen [52]. Such results, due to relatively low identity score, might represent mismatched identification within the specific genera, as most of MALDI-TOF microbial databases were developed and are still primarily utilized for a rapid epidemiological screening [51, 52].

The lack of identification of almost 70% of tested isolates with MALDI-TOF MS can be related to several factors, including relatively high differences in metabolic profile and protein composition of environmental strains in relation to reference microorganisms of collection or clinical origin, which are typically used for the construction of commercially available databases [53]. Changes in the metabolic profile of microorganisms due to symbiotic plant-bacteria relations can also influence the protein makeup [54], and in return could result in a mismatch or a lack of match with database reference mass spectra. Differences in identification rate could be due to the use of different MALDI-TOF systems and databases, among which the Microflex LT MALDI-TOF mass spectrometer (Bruker Daltonics, Germany) and the Biotyper database demonstrate a higher identification rate [55, 56]. For higher success rates of taxonomic identification, an enriched or dedicated database for AXIMA-iD Plus Confidence MALDI-TOF mass spectrometer (Kratos Analytical Ltd., Manchester, UK and Shimadzu Corporation, Kyoto, Japan) focused on the endophytic bacteria should be developed and tested [57–59].

Toubal et al. (2018) examined the identical plant sourced from two distinct areas in northern Algeria with the use of MALDI-TOF MS technology [60]. They identified 49 out of 57 isolates (85.96%), among which *Bacillus* sp. was the most abundant. Only strains belonging to the Enterobacteriaceae and Staphylococcaceae families were present in both plants. The most significant difference is the high abundance of *Pseudomonas* species detected in *U. dioica* L. from Poland presented in this study, in contrast to none found in the Algerian plant. The difference in identification rate is also notable, when comparing this study with Toubal et al. (2018a), which is 85.96% with ours 31.82%.

The dominance of *Bacillus* and *Pseudomonas* species among endophytic bacteria was also demonstrated by Mamarasulov et al. (2022) [61]. In their study, 11 of the 12 isolates obtained from the medical plant *Ajuga turkestanica* were identified as belonging to one of those genera by MALDI-TOF MS [61]. Furthermore, *Bacillus*,* Pseudomonas*, and members of the Enterobacteriaceae family were identified as endophytes in *Curcuma zedoaria* [62]. In the research by Wang et al. (2023), *Bacillus* and *Pseudomonas*, along with *Rhizobium* and *Microbacterium*, were detected in tissues collected from various *Populus* species [63]. Noticeably, the study demonstrated that the utilization of various growth conditions, including incubation temperature, different microbial media as well as growth period of tested cells were impacting the expression of ribosomal proteins and therefore abundance of mass spectra peaks [63]. This can directly impact the outcome of the MALDI-TOF MS identification process as important marker proteins can be absent or in concentrations too low for detection [63]. This is a crucial finding, showcasing that growth parameters cannot be simply omitted in MALDI-TOF MS identification workflow. On the other hand, the significantly increased number of samples needed to conduct optimization studies for each of analyzed strains hinders the main advantages of MALDI-TOF identification ‒ the reduced time, workload and costs of the analysis in comparison to other molecular or biochemical identification techniques. Of course, as the study of Wang et al. 2023 presented, there is a possibility of growth protocol optimization based on one selected strain. This method, however, to ensure high accuracy, requires prior knowledge of the main taxonomic groups of the newly acquired isolates or large in-house number of reference strains, and is only feasible for closely-related microbial collections of low taxonomic diversity with similar optimal growth conditions. Therefore, such approach seems more appropriate to apply for development of robust reference mass spectra databases, that will encompass various parameters of microbial cultivation, including growth requirements of endophytic bacteria.

The results of the present study align with previous reports, confirming the common occurrence of *Bacillus* and *Pseudomonas* as the common endophytic genera. However, the relatively low identification rate of isolates in this study may indicate a more diverse reservoir of endophytic microbiota and need for further standardization of MALDI-TOF MS identification process and database creation. This suggests that broader taxonomic resolution could potentially be achieved through the use of various growth conditions utilized for microbes propagation before identification, utilization of alternative MALDI-TOF workflows and databases, or the application of the supplementary complementary identification techniques.

### Metagenomic analysis

To delve deeper into the microbiome of Polish *Urtica dioica* L., genomic DNA was extracted from roots, stems, and leaves of all collected plants, following surface sterilization. Approximately 500 bp V3-V4 hypervariable regions of the bacterial 16 S rDNA gene were further amplified using extracted DNA as matrix. The amplicons were purified and sequenced in-house on the MinION platform, resulting in 947,210 quality-filtered reads combined. On average, nearly 70% of reads could not have been assigned to any known taxa using Kraken, with minimum 59% for WP_R sample and maximum 93% for NP_L. The overall abundance of unknown records may have various origins: poor quality of sequenced DNA and/or sequenced data, technical limitation of nanopore sequencing and Kraken2 classifications or very high proportion of unidentified organisms living in the isolation environment. Considering the attributes of nanopore amplicon sequencing and the recognized constraints of existing classifiers for long-read 16 S data, technical considerations may possibly play a role in the higher proportion of unassigned reads; hence, both biological and technical explanations are possible. Since DNA quality was measured using widely accepted spectrophotometric and fluorometric methods, and all quality assurance steps were done according to manufacturer recommendations we cannot fully exclude that sequencing - or classification-related factors influenced the proportion of unknown reads.

Since sequencing depth varied highly between samples, we have decided to rarefy the results and exclude samples with very low read count (represented by barcodes 1, 2, 7, 9 and 11). The rest of the samples are in a similar range (app. 60,000–90,000 reads), except for barcode 5, which greatly exceeded that number. As a result, observed richness loss in isolates NP_S and WP_R exceeds ~ 65% and is also observed in other isolates. The changes are most likely to influence richness, Pielou’s evenness, and Fisher’s alpha parameters. The results in Table 3 are derived from rarefied raw data. It is important to remember that diversity measures based on a single rarefaction run may be impacted by random sample effects. Repeated rarefaction might give more reliable estimates, although the general trends are still comparable. On average, the highest Shannon diversity index was observed for isolates from roots (NP_R − 3.58, WP_R − 2.94, EP_R − 3.48), but high values of the index can also be observed for NP_S and CP_L samples (3.53 and 2.92, respectively). While the root isolates can be characterized by high values of the Shannon index, isolates from either stem or leaf are not far behind, with their calculated index values being in the 1.9–2.3 range. High Shannon index values are paired with very high values of the Simpson Diversity index (1-D). The lowest Simpson’s diversity index (0.61), Shannon diversity index (1.54), and Pielou’s evenness (0.47), along the highest Berger-Parker index (0.60) suggest that microorganisms isolated from NP_L sample were dominated by some microbial species. The opposite observations can be made for NP_R characterized by the highest Shannon’s and Simpson’s diversity indices (3.58 and 0.94 respectively), and Berger-Parker index of 0.18 and Pielou’s evenness of 0.77 indicating higher diversity and more even microbial distribution. It is worth noting that sample NP_L also has the lowest overall rarefied richness and the highest unknown reads rate. Those values are not in sync with values for other leaf isolates. Values of the Berger-Parker index are low, in the range of 0.1–0.4, except for the aforementioned NP_L sample (0.60). At the same time, the calculated Pielou’s evenness indicates that the general shapes of the microbial communities in all other samples are similar, with some species being more abundant than others.

Testing calculated indices between tissues using the Kruskal-Wallis test yields nonsignificant p-values for α = 0.05 across the board and statistically significant if a more lenient α = 0.1 is assumed for comparison of Richness, Fisher’s alpha and Shannon’s index. In those cases post-hoc Dunn test detects a statistically significant difference (once again assuming more lenient α = 0.1) in medians only between leaves and roots. It is worth noting that the result of those tests should be considered with caution, as the nature of rarefaction is such that the set of observations is random, as well as the minimal number of biological replicates per tissue limits statistical power. Non-uniform distribution could cause fluctuations in calculated indices values and eventually a different outcome of the statistical test. Our work is more geared toward exploratory analysis of potential differences in microbiome environments with focus on extracting data on potentially industrially usable strains rather than full statistical analysis of microbial communities. Our analysis does not allow for definitive establishment of microbiome relationships in analysed tissues due to low statistical power of the tests, high potential of type I error, and the amount of unclassified reads. The calculated indices allow only for the insights into general trends and require further validation.

The most diverse environments here are roots with the highest overall Shannon’s and Simpson’s indices, as well as the lowest Berger-Parker index and Pielou’s evenness. It indicates that root environment is the most welcoming for a wide range of microbes, and that there are often no dominant genera. It is worth noting that richness in isolates from roots is higher than in leaves or stems. Microbial diversity in both stems and leaves seems to be lower than in roots with lower general richness and lower value of both Shannon’s and Simpson’s indices. At the same time Berger Parker index of isolates from leaves appears to be slightly higher than those from roots and stems, with Pielou’s evenness being lower in stems than in roots but even lower in leaves. Thus, it creates an image of less diverse microbial communities in both stems and leaves. This data is in line with results from many other researchers on plants’ microbial communities [64–66]. Many microorganisms can colonize the roots; however, further transportation into the upper part of the plant is not always possible. Also, there are different types of endophytes, and not all of them are strictly bound to the plant for all of their life cycle [12].

**Table 3:** α-Diversity indices across different samples from *Urtica dioica* L

Indices	CP_L	NP_R	NP_S	NP_L	SP_S	WP_R	WP_L	EP_R	EP_S	EP_L
Unknown counts	4,884	8,653	52,646	16,657	12,568	12,912	5,917	9,971	4,753	3,675
Total counts	6,814	12,213	74,259	17,890	17,162	21,660	6,836	13,370	7,351	4,378
Relative unknown	0.72	0.71	0.71	0.93	0.73	0.60	0.87	0.75	0.65	0.84
Shannon diversity index (H)	2.92	3.58	3.53	1.54	2.35	2.94	1.93	3.48	2.01	2.32
Simpson’s index (1-D)	0.89	0.94	0.95	0.61	0.84	0.89	0.76	0.93	0.77	0.81
Richness	76	105	95	26	48	80	34	107	39	49
Richness loss	32.32	47.09	68.58	29.41	44.83	62.90	5.13	44.39	55.22	0.00
Berger Parker index (BP)	0.18	0.18	0.11	0.60	0.26	0.27	0.40	0.19	0.34	0.34
Fisher’s alpha	21.65	34.19	29.61	5.31	11.66	23.24	7.46	35.14	8.90	11.98
Pielou’s evenness (J)	0.67	0.77	0.77	0.47	0.61	0.67	0.55	0.74	0.55	0.60

**Fig. 5:**
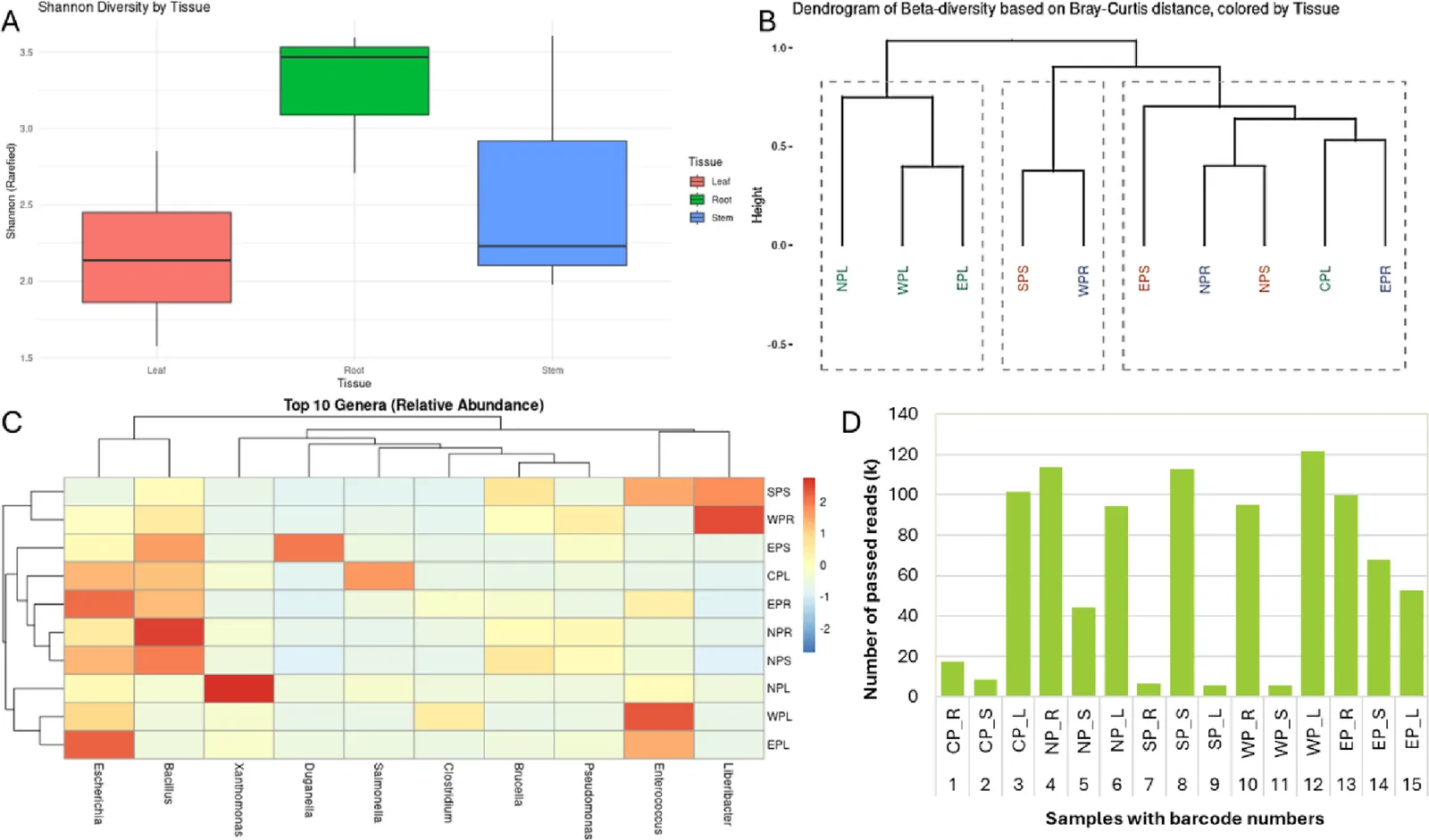
**A** Shannon diversity index by tissue; **B**. Clustering of samples based on Bray-Curtis distance coloured by tissue of origin; **C** Relative abundance of the 10 genera with the highest average abundance across all samples. Abundance values have mean score of 0 and standard deviation of 1; **D** Number of passed reads for each sample

**Fig. 6:**
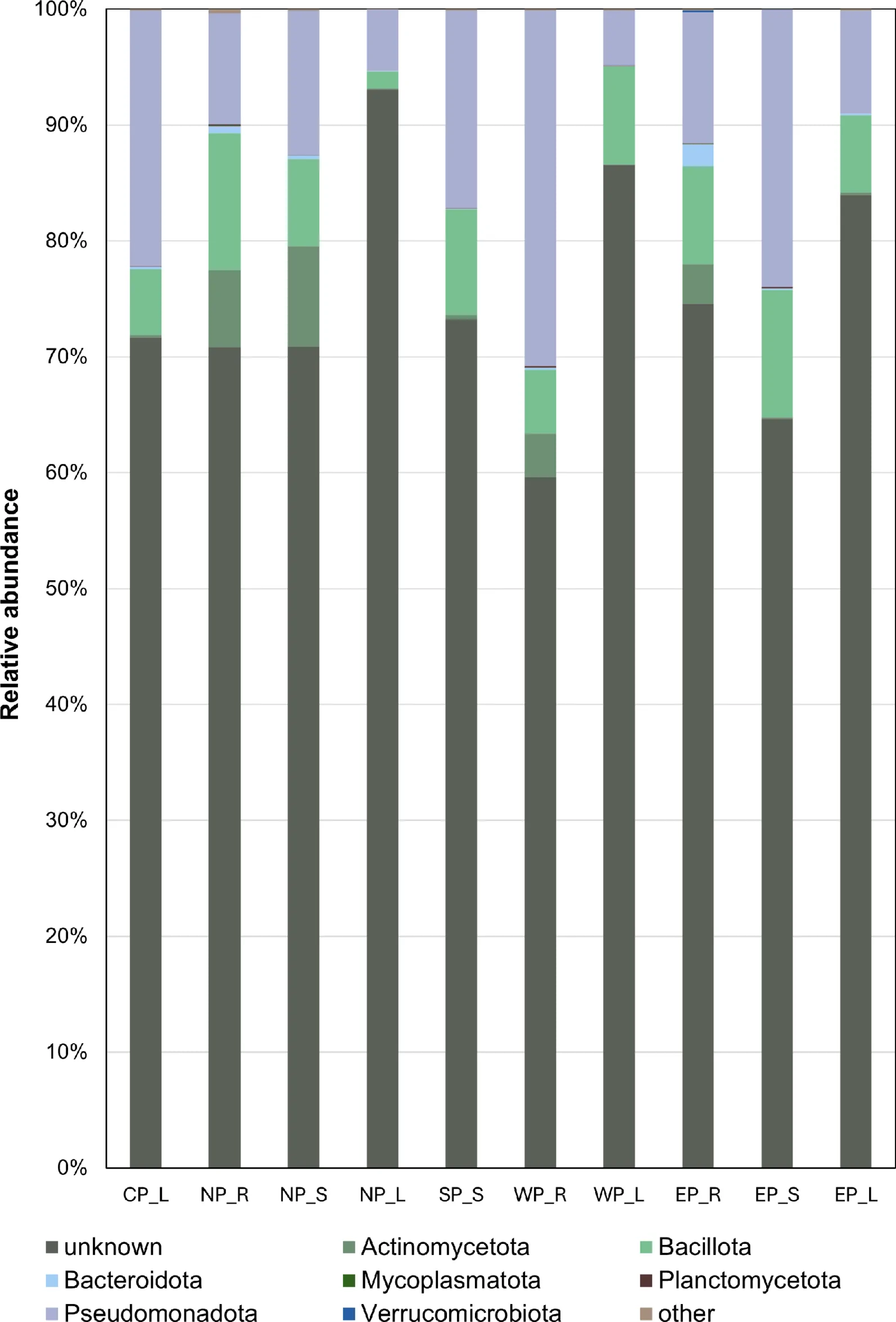
Barplot of the most abundant taxa at the phylum rank in all the samples

Following the removal of any host sequences that mapped against a provided host reference (in that case chloroplast and mitochondrial sequences from *Urtica dioica* L.), the taxonomic classification of the sequences and estimation of the abundance were conducted. Reads were classified into 27 phyla, 59 classes, 136 orders, 256 families, and 577 genera. First, the pattern of diversity within the phylum level of the plant samples was studied (Fig. 6, Table S1).

Any remaining taxa have collapsed under the ‘other’ category to facilitate the visualization. The y-axis indicates the relative abundance of each taxon in percentages for each sample.

As visible in Fig. 6, all plant samples contained the vast majority of sequences, which could not be classified into known bacterial phyla. The number varies from 60% (WP_R) to 93% (NP_L). This comes as no surprise, as currently, we can grow only approx. 1–2% of microorganisms present in the environment under laboratory conditions [67]. Three phyla are present in all samples – Actinomycetota, Bacillota and Pseudomonadota; however, their quantity varies. It probably depends on conditions occurring in the place of isolation (weather, soil composition and pH, temperature, etc.). Those phyla are the most common across many plants [68, 69]. Samples extracted from leaves can also be less diverse than roots, which is related to the abundance of putative passenger endophytes from the rhizosphere and the motility of the bacteria.

**Fig. 7:**
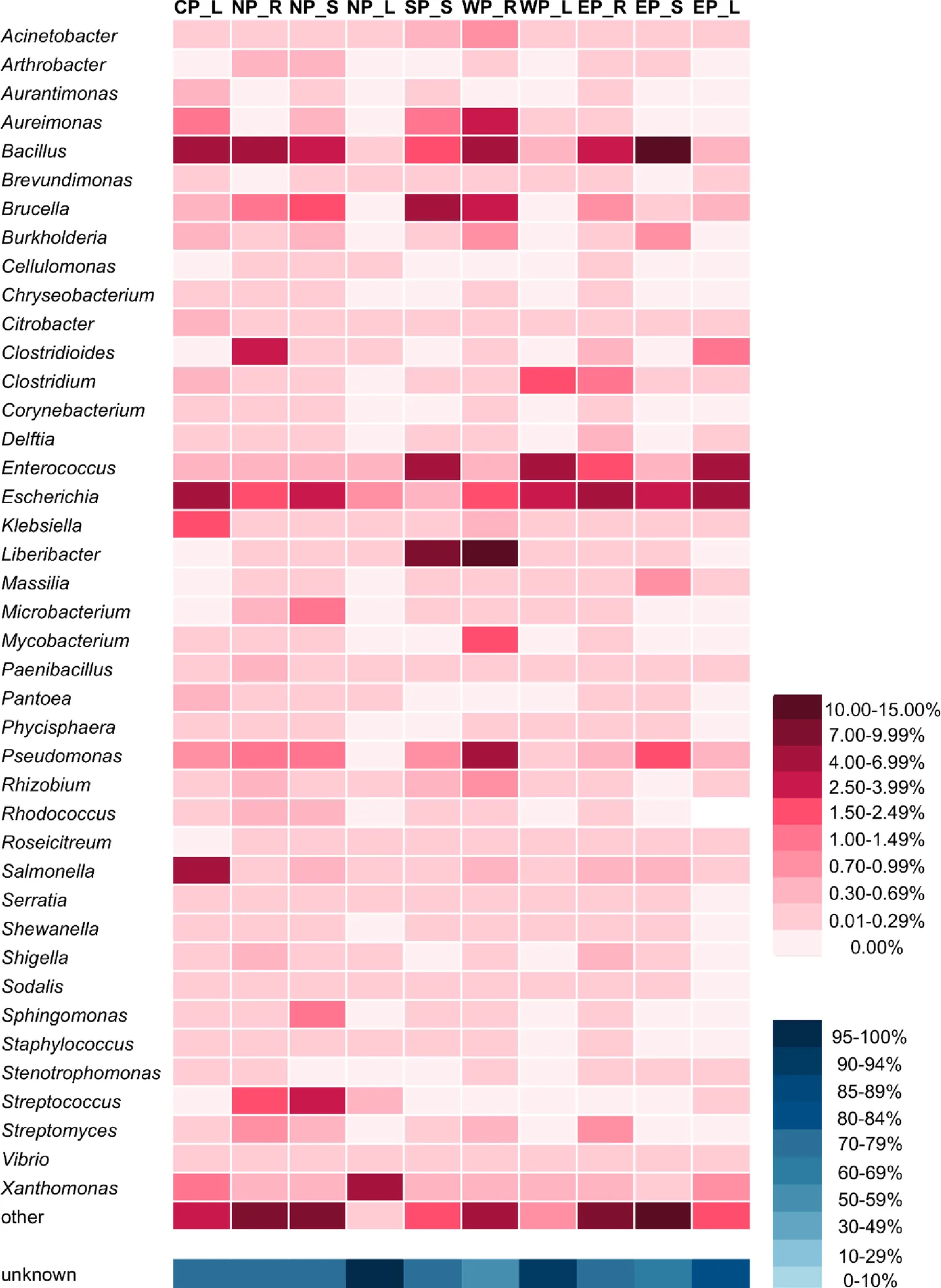
Heatmap showing relative abundance of the most abundant taxa at the genus rank in all the samples. Any remaining taxes have collapsed under the ‘other’ category to facilitate the visualization

The most abundant taxa at the genus rank were studied in detail (Fig. 7). The percentage of reads assigned to various genera across all samples, with the most common genera being highlighted, is summarized (Table S2).

From all of these genera, MALDI-TOF MS analysis revealed the presence of nine *Bacillus* spp. strains across all plant samples, which is consistent with metagenomic data. Also, from all plant samples, except for CP, members of *Pseudomonas* spp. were isolated and identified. It heavily depends on the culture media and databases used, as we discussed before.

While comparing the microbial communities of *Urtica dioica* L. from Poland with those from different regions, it is evident that most research reports a dominance of *Bacillus* species. In our previous study [26], two *Bacillus* species with potential for biosurfactant and polyphenolic compounds production were identified. Additionally, *Bacillus* and *Paenibacillus* strains from Moroccan *Urtica dioica* L. were examined for their significant antagonistic activities against plant pathogens [70, 71]. Common culturable genera found in plants isolated from various regions include *Arthrobacter*,* Bacillus*,* Brevundimonas*,* Enterobacter*,* Enterococcus*,* Escherichia*,* Methylobacterium*,* Paenibacillus*,* Pantoea*,* Paracoccus*,* Staphylococcus*,* Stenotrophomonas*, and *Streptomyces* [60, 72, 73]. For instance, *Azotobacter* was found only in *Urtica dioica* L. from Serbia [72], while *Achromobacter*, *Cellulosimicrobium*, *Micrococcus*, and *Starkeya* were identified solely in plants from the Czech Republic [73].

For our greatest knowledge, our study is the first metagenomic study on *Urtica dioica* L. plant; all of the above-mentioned research studied only culturable strains from the microbiome. Thus, we could “reach deeper” into the actual microbiome of *Urtica dioica* L. In the plant studied here, certain genera were identified in all samples, which are *Acinetobacter*, *Bacillus*, *Citrobacter*, *Enterococcus*, *Escherichia*, *Klebsiella*, *Paenibacillus*, *Salmonella*, *Vibrio*, and *Xanthomonas*. All of them were present in every sample tested in this study, yet they had not been reported in previous studies on endophytes from *Urtica dioica* L.

**Fig. 8:**
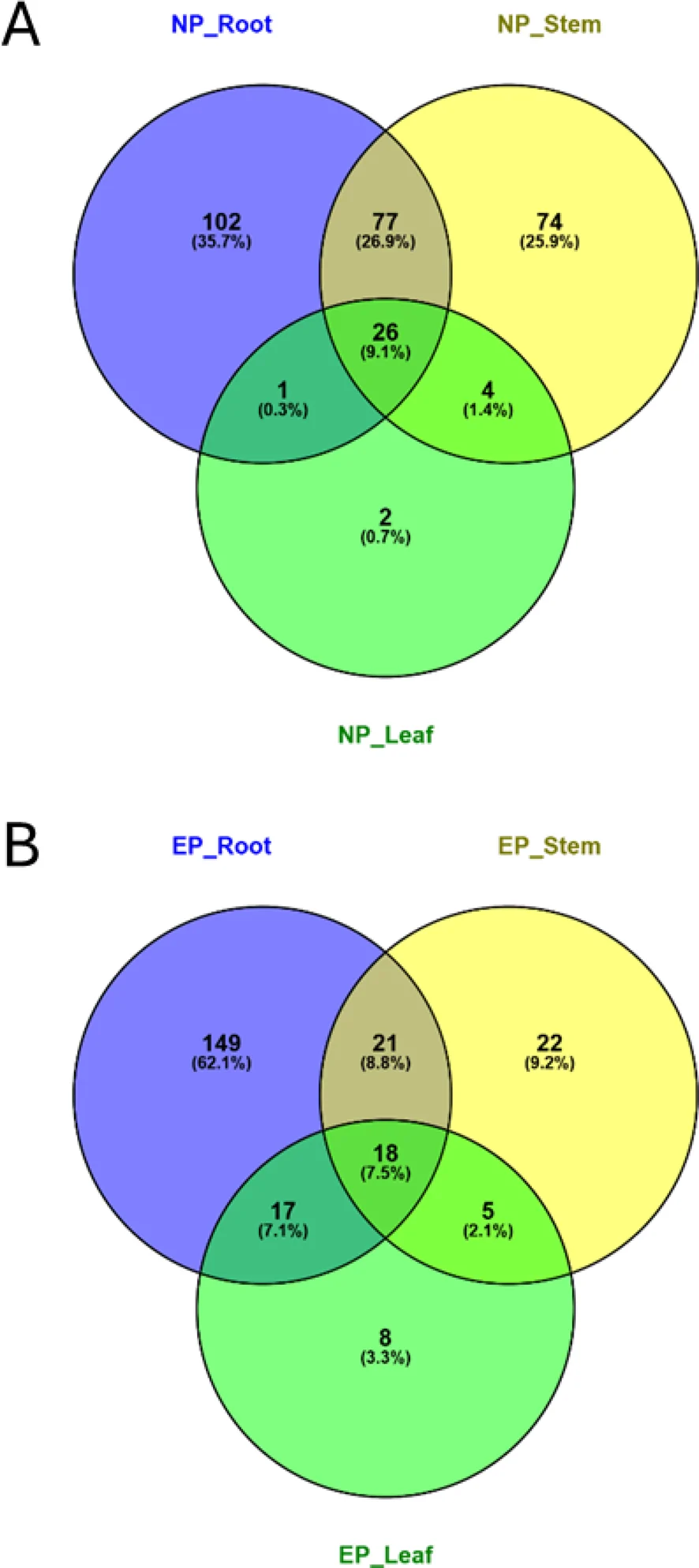
Venn diagrams of endophytic bacterial communities across tissues are shown for the whole plants isolated from two regions - North (**A**) and East (**B**). Bolded numbers on the graph represent an abundance of bacterial genera (genera found in ≥ 0.1%)

Samples from the Northern Plant (NP) and Eastern Plant (EP) were studied in greater detail, as we obtained sufficient sequencing reads from all three tissue types - root, stem, and leaf (Figure S1). In both plants, leaf community remains the most elusive, while other tissues possess slightly higher amounts of identified sequences. As stated before, ten genera were detected in all samples, although their relative abundances differed considerably between tissues and locations. Each plant and each sample even has its specific genus of bacteria, for instance, *Duganella* spp. representatives in EP’s stem or *Bifidobacterium* spp. in NP’s roots and stem. After assessing similarities in samples, the differences were highlighted (Fig. 8).

Venn diagram shows shared and unique endophytic bacterial taxa among tissues of *Urtica dioica* L. from samples isolated from North and East. The NP sample exhibited greater diversity among bacterial genera, identifying 286 specific genera in total (Fig. 8A). In contrast, the EP sample revealed 240 genera of endophytic bacteria (Fig. 8B). Within the NP sample, a significant majority (88.5%) of these genera were localized in the root and/or stem. In the leaf tissues, the diversity was significantly lower, with only 2.4% of specific genera identified in leaves alone, in combination with stems, or in combination with roots. Furthermore, 9.1% of the genera were detected across all tested tissue types, indicating some level of consistency in the bacterial community present in the NP sample. These results are consistent with previous diversity analyses that indicated limited differences between tissues. On the other hand, the unique genera of each tissue separately accounted for 62.3%. The high similarity of the same genera in both roots and stems (26.9%) of the NP plant indicates that endophytic bacteria were most likely migrating within the plant; nevertheless, alternative hypotheses, such as distinct colonization episodes or common soil-derived inoculum, cannot be entirely disregarded.

On the contrary, EP showed significantly higher diversity among each single tissue (74.6% of all found genera were classified for only leaf, stem, or root individually). Additionally, EP sample showed a lower abundance of genera found in the whole plant (7.5% of genera were shared in each tissue - leaf, stem and root). The EP sample had significantly higher diversity of genera found only in roots (62.1%) and leaves (3.3%), as well as found in both of those tissues (7.1%). EP showed also a lower abundance of unique strains for stem (9.2%) and both stem and root (8.8%).

**Fig. 9:**
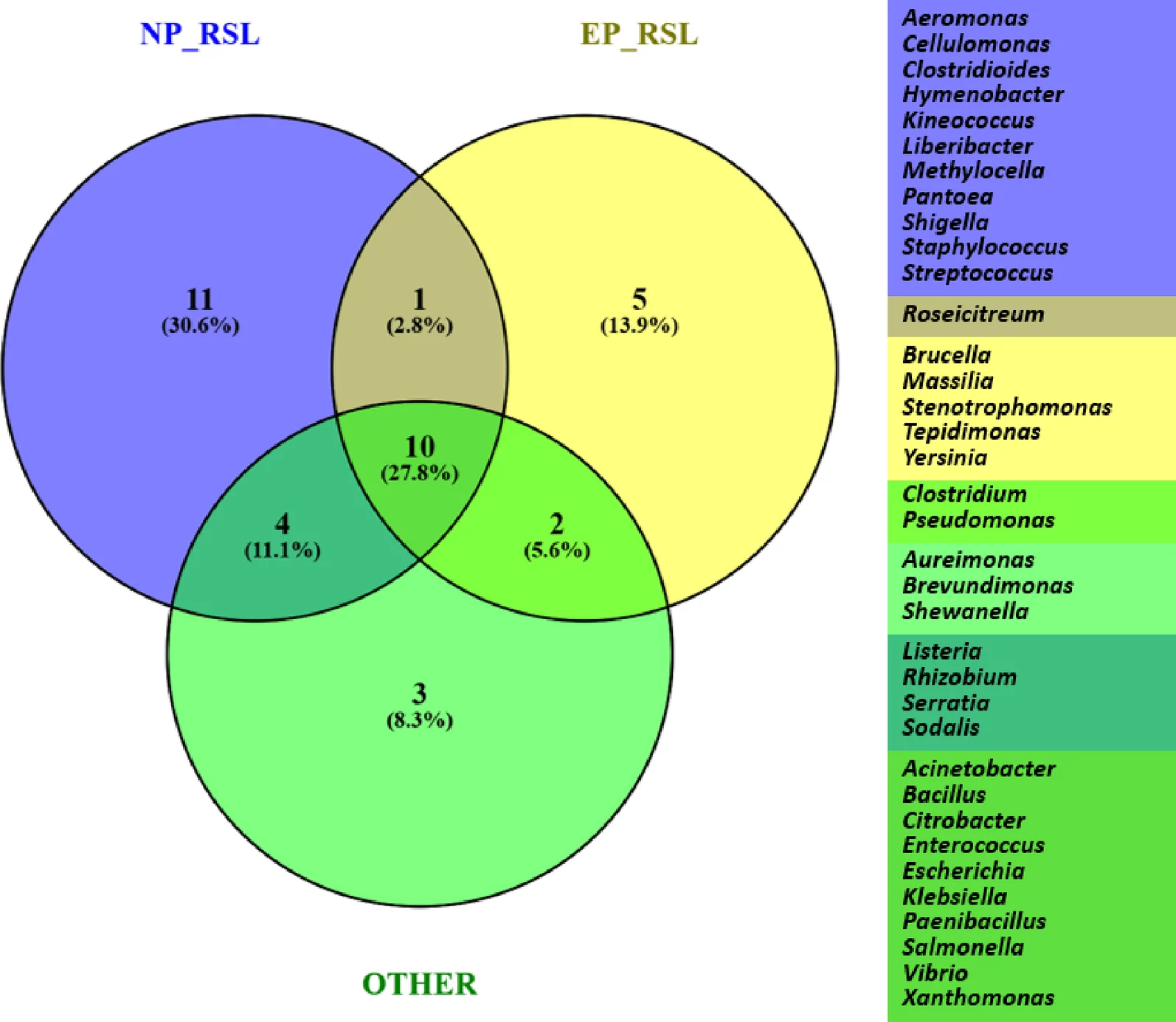
Venn graph – comparison of genera which are present in every tissue (number of shared genera present in root, stem and leaf) for NP and EP compared to all other samples. Such analysis was performed as NP and EP were the only samples for which OTU for every tissue was obtained. The genera shown on the right, presented on corresponding colors from the Venn diagram, show the exact composition of each Venn subgroup

Figure 9 compares the genera of plants obtained from different parts of Poland and indicates the specific genera for each indicated region (NP, EP or others tested). In case of NP and EP, only genera shared among every tested tissue were counted, while for others, every genus shared by Central, Southern and Western plants was counted as those plants didn’t generate enough data for every tissue to consider them as whole plants. It is shown that ten genera are putatively recurrent taxa of *Urtica dioica* L. plant, no matter the region of origin. All of the shared genera were previously known as endophytes [74–83].

Members of the genera *Bacillus* and *Paenibacillus* are among the most extensively studied endophytic bacteria, isolated from various plants across the world [84, 85]. Endophytic *Bacillus* spp. provides multiple benefits to their host plants, including protection against pathogens, production of phytohormones and hydrolytic enzymes, and nutrient solubilization. The formation of spores able to resist hostile conditions is one of the main reasons for *Bacillus* use in agriculture [86]. Moreover, *Bacillus* endophytes produce a variety of bioactive compounds such as biosurfactants [87], polyphenols [26], and siderophores [88]. Their genomes encode extensive biosynthetic gene clusters responsible for the production of diverse metabolites [12, 89]. The genus *Paenibacillus* was previously classified as *Bacillus*, for example genera such as *B. glucanolyticus* or *B. alginolyticus* were redefined based on the whole genome sequencing [90]. They possess similar broad range of plant promoting traits to *Bacillus* spp. For instance, endophytic isolates representing *Paenibacillus polymyxa* species (BR20, SeR8) from wheat exhibited complementary antagonistic activities against two phytopathogens, *Xanthomonas translucens* and *Fusarium graminearum*, respectively [91]. Another *P. polymyxa* LMG27872 was shown to inhibit the major pest in tomato production, nematode *Meloidogyne incognita* [82]. Not only *P. polymyxa* is frequently isolated, but also *P. dendritiformis* and *P. amylolyticus*, both demonstrating plant growth-promotinh or microbial potential [92, 93]. Several novel *Paenibacillus* taxa have been reported from plant roots and nodules, including *P. medicaginis*, *P. endophyticus*, *P. borealis* and *P. lupini*, highlighting the broad ecological distribution and functional diversity of this genus [94–96].

*Acinetobacter* is another beneficial genera found as endophytic, including medicinal plants. For instance, *Acinetobacter johnsonii* was identified among the two culturable endophytic bacteria from seeds of *Tamarindus indica* L. Experiments revealed a stimulatory effect of seeds’ exudates, rich in indole-like substances, which were also present in bacterial culture filtrate [97]. On the other hand, *A. schindleri* from traditional Chinese herbal plant *Pseudostellaria heterophylla* demonstrated high degradation activity to toxins of common fungal pathogen *Fusarium graminearum* [98]. However, some members of its genus, such as *A. baumannii*, are known human pathogens capable of biofilm formation and antibiotic resistance. Interestingly, endophytic isolate from papyrus was reported to produce biosurfactants with antibiofilm activity against *(A) baumanii* [99]. Thus, this demonstrates the complexity of possible microbial interactions within the plant, where synergistic and antagonistic relationships coexist. Although *Klebsiella* species are primarily recognized as opportunistic pathogens in humans, recent studies have emphasized their underexplored agricultural potential. Da Silva Bandeira and others (2025) conducted a meta-analysis of endophytic *Klebsiella* and their potential benefits for plant growth and suppressing [100]. Notably, *K. pasteurii* BDA134-6 isolated from African rice not only very effectively colonized wheat but also improved their response to drought stress by mainly increasing antioxidant enzyme activities [101]. *K. pneumoniae* has also been proposed as a potential biofertilizer owing to its multiple plant growth-promoting traits, alongside *(B) subtilis* and *B. amyloliquefaciens* [77]. Similarly, *K. aerogenes* HGG15 improved mulberry growth under drought stress, while showing no toxicity against silkworms, the plant’s primary consumer [102].

While *Enterococcus* and *Escherichia* are more commonly associated with animal microbiomes, they can also establish endophytic relationships with plants. Inoculation of tomato and Bermuda grass with GFP-tagged *E. coli* significantly enhanced root development, presumably due to indole-3-acetic acid (IAA) production [83]. Naturally occurring plant *E. coli* endophytes have been isolated from oil palm, where they contribute to plant growth and colonize other host upon inoculation [103]. Interestingly, Svec and others (2012) isolated few *Enterococcus* spp. from various plant flowers, including herbs such as *Carduus* and *Thymus*, leading to the proposal of *Enterococcus plantarum* sp. nov [104].

*Salmonella* spp., although primarily known as human pathogen, can survive and colonize plant tissues as well. Contamination of vegetables and fruits with human pathogen bacteria occurs through irrigation water or soil, leading to internalization within plant tissues [105]. Their persistence in agricultural soil depends heavily on both soil type and plant species [75].

The genus *Xanthomonas* comprises well-known phytopathogens responsible for diseases such as leaf blight, leaf spot, and black rot in numerous crops [106, 107]. Despite their pathogenicity, *Xanthomonas* has been a valuable model for studying bacterial virulence, host adaptation, and biofilm formation, primarily due to the production of the polysaccharide xanthan, a key biofilm component with industrial applications [108]. While typically soil-associated, some *Xanthomonas* species are non-pathogenic [109] and may even protect plants from pathogenic strains. Members of the next genus, *Vibrio* spp., are primarily aquatic bacteria, common in marine and estuarine environments. Although rare, endophytic *Vibrio* species have been detected, such as isolates from *Salicornia europaea*, a halophytic plant inhabiting saline soils [110].

Finally, *Citrobacter* spp., commonly associated with opportunistic infections in humans, have also been identified as endophytes. *Citrobacter freundii* RPT, isolated from the arsenic-hyperaccumulating fern *Pteris vittata*, exhibited high arsenic tolerance and removal efficiency [111]. Similarly, *C. freundii* AYS58, an endophyte from maize roots, possesses genes related to phosphate and zinc solubilization [79].

However, among the rest of the results, new and not described yet as endophytes were also detected *– Listeria*,* Clostridioides*,* Liberibacter and Roseicitreum*. All of the genera found in plants other than Northern and Eastern ones were growth-promoting endophytes. Li et al. (2017) identified *Aureimonas* spp. as siderophore-producing bacteria [112]. *Brevundimonas diminuta* and *B. vesicularis* isolated from peaches, showed plant growth-promoting potential based on their indoleacetic acid (IAA) production, nitrogen fixation, and solubilization of phosphate [113]. *Shewanella* spp. could enhance stress tolerance in plants, as described by Manjunatha et al. (2022), where effect of *Shewanella putrefaciens* on pearl millet was tested under drought stress by stimulating the production of ABA and IAA in plants [114] .

### Analysis of pigment-producing endophytic bacterial strain

**Fig. 10:**
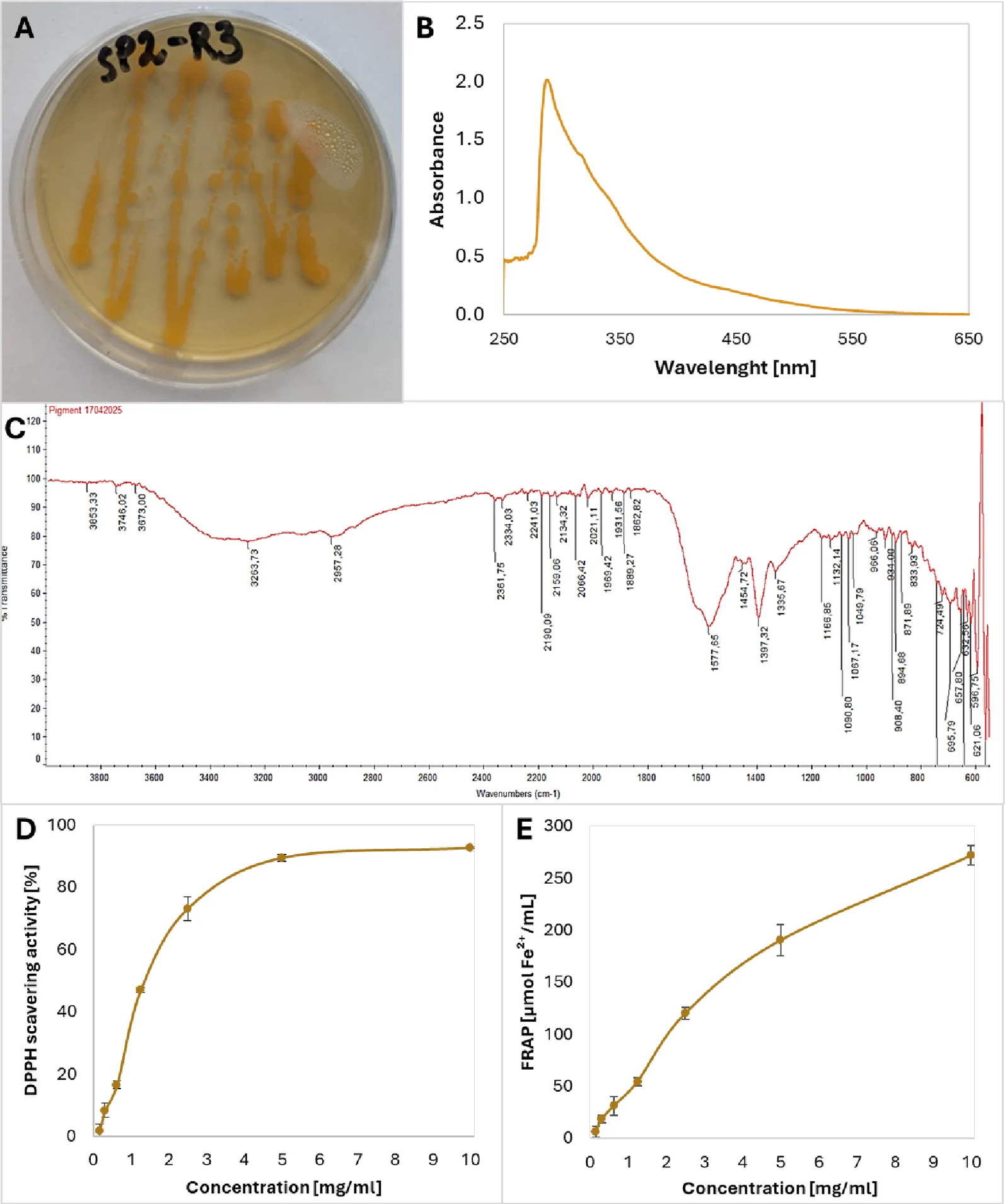
Characteristics of pigment obtained from strain SP2-R3. **A** The endophytic bacterium grown on NB agar medium; **B** UV-Vis spectra of extracted pigment; **C** FTIR spectra of extracted pigment; **D**–**E**, Antioxidant activity assays for SP2-R3 pigment **D** scavenging of DPPH radicals and (**E**) scavenging of ferric reducing antioxidant power (FRAP)

Some of the strains showed putative pigment production while growing on agar plates. A quantitative assessment of pigment synthesis was conducted using the isolated pure bacterial cultures. Among the isolates, strain SP2-R3 (Fig. 10A) exhibited the most intense orange pigmentation and rapid growth; thus, it was chosen for a detailed analysis as a potential pigment producer.

Synthetic pigments are widely used in various industries due to their affordability, vibrant colours, and high stability. However, their application in sectors such as the food industry is linked to potential health concerns, including allergenicity and carcinogenicity. To address the growing demand for safer colouring alternatives, microbial pigments could serve as an alternative [115]. Microbial pigments have several advantages, including high production potential due to rapid growth rates, ability to utilize low-cost substrates, and consistent production under controlled conditions [116]. Moreover, they are less toxic, more biodegradable, and may offer beneficial effects like antioxidant and anticancer properties [117]. Additionally, their biosynthesis can be enhanced through genetic engineering to improve yield and tailor pigment characteristics [118].

*Urtica dioica* contains a range of pigments, including chlorophylls a and b, quinones such as vitamin K, and various carotenoids such as neoxanthin, violaxanthin, lutein and its derivatives, as well as β-carotene [8, 119]. Endophytic bacteria associated with plants can produce a similar spectrum of secondary metabolites to those found in their host species [12, 120]. For instance, the endophytic *Serratia marcescens* strain VIT-PTS, isolated from the leaves of *Beta vulgaris*, synthesizes the red tripyrrole pigment prodigiosin [121]. According to the study done by Meyer (1978), *Pseudomonas fluorescens* produces yellow-green pigment – pyoverdine [122]. It is a siderophore that transports Fe^3+^ ions. This species also produces pyrroloquinoline quinone, which is a plant growth promotion factor [123, 124]. While specific studies on PQQ’s effects on common nettle are lacking, the general plant growth-promoting properties of PQQ suggest potential benefits for nettle growth.

Other endophytic bacteria have also demonstrated pigment production with potential bioactive properties, such as carotenoids. Endophytic *Citricoccus parietis*, isolated from medicinal plant *Calotropis procera*, has been reported to produce β-carotene [125]. *Micrococcus luteus*, isolated from *Avicennia marina*, produces a yellow pigment. This pigment is likely a carotenoid with antioxidant activity, as indicated by an IC₅₀ value of 2.29 µg/mL in the DPPH assay [126]. Pigment production was visually assessed based on colony morphology during cultivation. The strain selected for further study was chosen due to its pronounced pigmentation.

Based on an extensive literature review, an appropriate extraction method was selected for isolating pigments belonging to the quinone and carotenoid classes. Carotenoid and isoprenoid quinones extraction efficiencies are known to be dependent on both pigment and solvent polarity. Therefore, acetone and methanol were selected for co-extraction of pigments, which allowed extraction of non-polar pigments (better extracted with acetone) and polar pigments (preference towards methanol) [38, 127, 128]. The extraction yielded a pigment concentration of 51.18 ± 5.44 mg per gram of wet bacterial biomass, indicating effective recovery. UV-Vis spectra show main absorbance peak at 287 nm. UV-Vis spectroscopic analysis of the extracted pigment (Fig. 10B) supports its classification within the quinone family. The spectral profile closely resembles that of hydroquinone or naphthoquinones and their derivatives [129–131], thus confirming the initial hypothesis regarding the pigment’s quinone-like chemical nature.

**Table 5:** FTIR peak assignments obtained pigment from the bacterial strain SP2-R3

Bond	Type of bond	Transmitation cm^− 1^	Appearance
O-H	Alcohols, phenols, water	3263,73	Broad
C-H	Alkyl, especially methyl	2957,28	Weak
NH_2_	Amino group	1577,65	Strong
C-H	Alkyl	1397,32	Strong
C-O	Aromatic streches	1090,80	Medium

Using the FTIR technique enabled to obtain the spectra of the raw extract and the identification of the functional groups present in the isolated pigment (Fig. 10C). The results of the FTIR analysis support the findings of the UV-VIS examination of the acquired pigment (Table 5). The absorption band at 3393 cm − 1 corresponds to the O–H stretching vibration of the hydroxyl group at around 3264 cm − 1. The figure displays the aliphatic C-H stretching vibrations at approximately 2957 cm⁻¹. The band at around 1577 cm-1 is linked to the absorption bending vibration of the amino group (NH_2_). The spectrum indicated the presence of bending vibrations of the C-H bond (~ 1397 cm-1) and vibrations related to the C-O bond (~ 1091 cm-1). Carbonyl groups (C = O) were not identified. The results obtained indicate the pigment in question is a member of the quinone group. The FTIR spectrum exhibits a high degree of similarity to the spectra of hydroquinone, naphthoquinones, or anthraquinones [132–134].

In order to evaluate the biotechnological potential of the pigment produced by strain SP2-R3, its antioxidant activity was examined. The determination of antioxidant activity for microbial pigments is essential due to their huge potential as natural alternatives to synthetic antioxidants, widely distributed in food, pharmaceutical, and cosmetic industries. Such pigments frequently exhibit strong radical scavenging properties and can mitigate oxidative stress-related damage. Moreover, bacterial production offers sustainability and scalability advantages compared with plant or animal-based antioxidant sources [135, 136].

The DPPH assay is a widely used method for evaluating the free radical scavenging ability of antioxidants. DPPH is considered a stable free radical that exhibits a deep violet color in ethanol. Upon interaction with an antioxidant, DPPH accepts an electron or hydrogen atom, leading to decolorization of solution, which can be quantitatively measured by a decrease in absorbance at 517 nm [137]. DPPH activity is shown as percent of scavenging activity or as IC50 value, where lower IC50 values are desired as they equal more efficient antioxidants. For pigment extract obtained from SP2-R3 strain (Fig. 10D), IC50 = 1.40 ± 0.06 mg/ml and it showed 92.67 ± 0.31% scavenging activity for highest tested concentration of 10 mg/ml. Although this value is significantly lower than the DPPH activity of standard ascorbic acid, which demonstrated a 95.93 ± 0.65% scavenging activity at the lowest tested concentration of 156.25 µg/ml, the SP2-R3 pigment still has potential for further investigation and offers additional functional possibilities.

Another commonly used method to determine antioxidant potential is Ferric Reducing Antioxidant Power (FRAP) assay. The FRAP assay chemical reaction is based on the capacity of a Fe^3+^ complex reduction from colorless complex into a blue-colored one [Fe^2+^(TPTZ)_2_]^2+^ in acid medium [40]. Color changes are measured at a spectrophotometer at 593 nm. The antioxidant capacity is typically expressed in terms of µmol Fe²⁺ equivalents per gram or milliliter of sample. In this assay (Fig. 10E), the highest tested concentration of 10 mg/ml yielded a FRAP activity of 271.67 ± 9.27 µmol Fe²⁺/mL, which is lower than the corresponding value for ascorbic acid, recorded at 33642.24 ± 50.71 µmol Fe²⁺/ml. Nonetheless, presented pigment extract demonstrates potential scavenging activity.

### Identification of pigmented strain based on 16 S rDNA

**Fig. 11:**
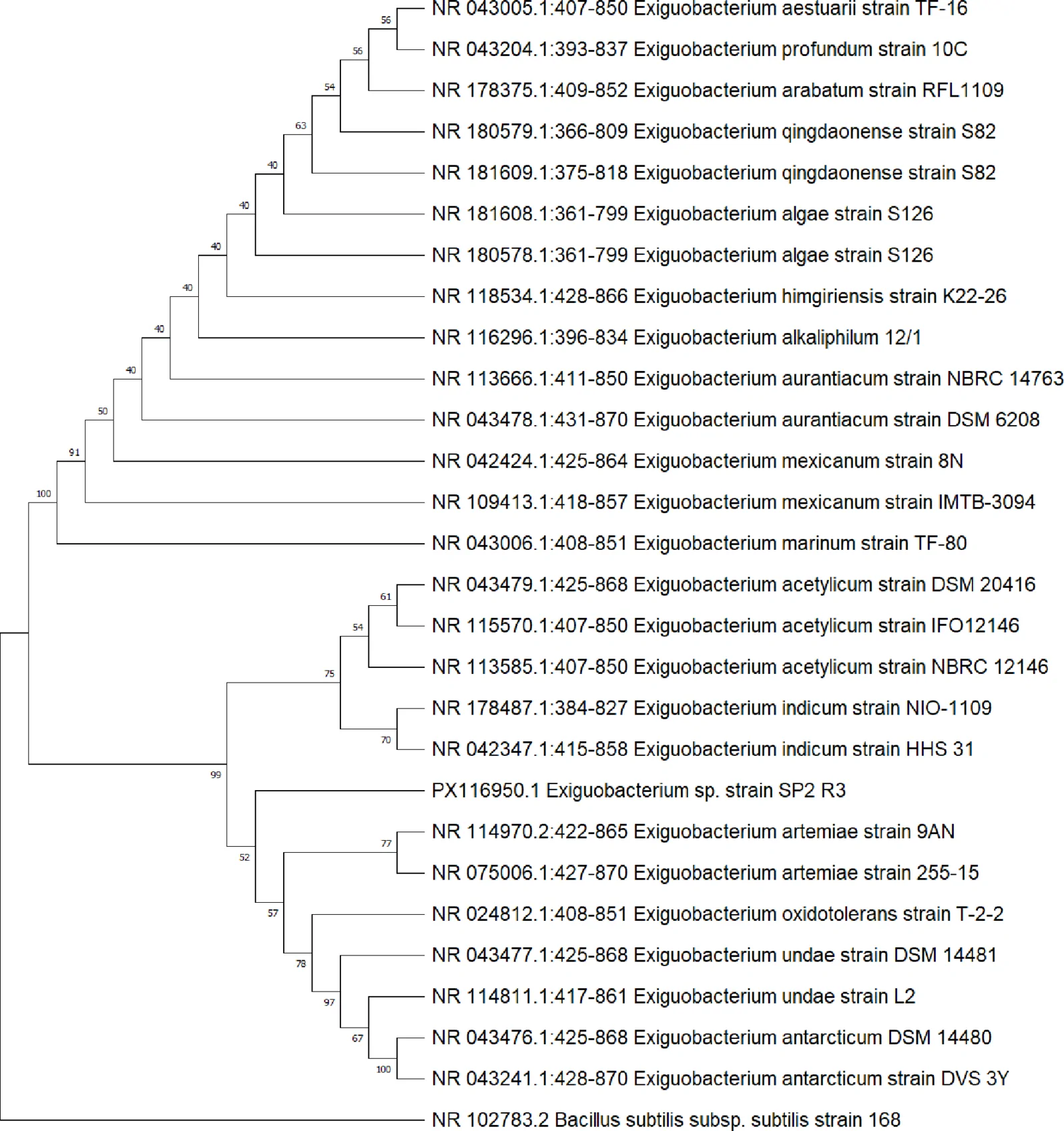
Neighbour joining phylogenetic tree of *Exiguobacterium* strains including strain SP2_R3 based on their 16 S rDNA gene sequences

The best pigment producer was subjected to 16 S rDNA sequencing (V3-V4 fragment) for identification. The analysis revealed a close relationship with *Exiguobacterium* species (Fig. 11). The sequence was deposited in the NCBI database under the accession no. PX116950.1.

*Bacillus subtilis* subsp. *subtilis* strain 168 was used as outgroup. The bootstrap consensus tree is inferred from 1000 replicates, branches corresponding to partitions reproduced in less than 50% bootstrap replicates are collapsed. The percentage of replicate trees in which the associated taxa clustered together in the bootstrap test (1000 replicates) are shown next to the branches. The evolutionary distances were computed using the p-distance method and are in units of the number of base differences per site.

## Conclusions

The present study provides preliminary insights into the diversity of endophytic bacteria residing in *Urtica dioica* L. gathered from five climatic regions of Poland. Through the integration of culture-dependent and metagenomics, variations in microbial community composition among plant tissues (roots, stems, leaves) and across geographic areas were elucidated. Roots exhibited the greatest microbial richness and diversity, reinforcing the notion that numerous endophytes derive from the rhizosphere prior to invading interior plant tissues.

Despite the detection of 10 bacterial genera across all samples, which may represent a putative core microbiome of *U. dioica*, these taxa accounted for less than 30% of all identified genera. However, this observation should be interpreted with caution given the limitations of taxonomic resolution in metagenomic classification of plant-associated microbiomes, as a substantial fraction of reads remained unclassified. Future studies employing improved reference databases and complementary classification approaches will be necessary to refine taxonomic assignment and to validate the stability of the proposed core microbiome. This discovery suggests that the endophytic community is significantly influenced by external environmental variables, such as local soil conditions and climate. This heterogeneity underscores the necessity for future studies to investigate how environmental factors affect the assembly and adaptive functions of endophytes inside their plant hosts.

Culture-based isolation verified by the existence of many genera recognized for their plant growth-promoting or biocontrol capabilities, including *Bacillus*, *Pseudomonas*, *Escherichia*, and *Enterococcus*. Several isolates demonstrated the ability to produce color. Strain *Exiguobacterium* spp. SP2-R3 generated a quinone-related compound class with considerable antioxidant activity. Detailed chemical characterization of these pigments and metabolites, including structural elucidation, will be required to confirm their identity and evaluate their applicability. These bacteria may provide a significant supply of natural colors and bioactive metabolites, presenting sustainable alternatives to manufactured chemicals in medicinal, cosmetic, and agricultural sectors.

Overall, this work enhances current knowledge of *U. dioica* endophytic populations and signifies the importance of integrating cultivation with metagenomic analysis. Further research should focus on improving taxonomic resolution of plant-associated microbiomes, validating the existence and stability of the proposed core microbiome, and performing comprehensive chemical characterization of bioactive compounds. Such studies will be essential to fully assess the ecological roles and biotechnological potential of these endophytes and their metabolites. Also, additional research is necessary to clarify the processes connecting environmental influences on endophyte composition, and to assess the functional potential and use of these microbes and their metabolites in biotechnology and agriculture.

## Supplementary Information

Below is the link to the electronic supplementary material.

Supplementary Material 1. Table S1. Abundance table for the phylum rank. Table S2. Abundance table for the genus rank. Figure S1. Diversity of endophytic bacteria found in samples NP (A-C) and EP (D-F) divided into tissues: root (A, D), stem (B, E), leaf (C, F).

## Data Availability

Data is provided within the manuscript or supplementary files. Generated datasets are available upon reasonable request to the corresponding author.

## References

[1] Taylor K. Biological flora of the British Isles: Urtica dioica L. J Ecol. 2009;97:1436–58. 10.1111/J.1365-2745.2009.01575.X.

[2] Grosse-Veldmann B, Weigend M. The geometry of gender: hyper-diversification of sexual systems in Urtica L. (Urticaceae). Cladistics. 2018;34:131–50. 10.1111/CLA.12193. 34641638

[3] Devkota HP, Paudel KR, Khanal S, Baral A, Panth N, Adhikari-Devkota A, et al. Stinging Nettle (Urtica dioica L.): Nutritional Composition, Bioactive Compounds, and Food Functional Properties. Molecules. 2022;27:5219. 10.3390/MOLECULES27165219. PMC9413031 36014458

[4] Kasouni AI, Chatzimitakos TG, Stalikas CD, Trangas T, Papoudou-Bai A, Troganis AN. The Unexplored Wound Healing Activity of Urtica dioica L. Extract: An In Vitro and In Vivo Study. Molecules. 2021;26:6248. 10.3390/MOLECULES26206248. PMC8540079 34684829

[5] Taheri Y, Quispe C, Herrera-Bravo J, Sharifi-Rad J, Ezzat SM, Merghany RM, et al. Urtica dioica-Derived Phytochemicals for Pharmacological and Therapeutic Applications. Evidence-Based Complement Altern Med. 2022;2022:4024331. 10.1155/2022/4024331. PMC8894011 35251206

[6] Bhusal KK, Magar SK, Thapa R, Lamsal A, Bhandari S, Maharjan R, et al. Nutritional and pharmacological importance of stinging nettle (Urtica dioica L.): A review. Heliyon. 2022;8:e09717. 10.1016/J.HELIYON.2022.E09717. PMC9253158 35800714

[7] Namazi F, Bordbar E, Bakhshaei F, Nazifi S. The effect of Urtica dioica extract on oxidative stress, heat shock proteins, and brain histopathology in multiple sclerosis model. Physiol Rep. 2022;10:e15404. 10.14814/PHY2.15404. PMC9350467 35924324

[8] Jan KN, Zarafshan K, Singh S. Stinging nettle (Urtica dioica L.): a reservoir of nutrition and bioactive components with great functional potential. J Food Meas Charact. 2017;11:423–33. 10.1007/S11694-016-9410-4/TABLES/3.

[9] Rutto LK, Xu Y, Ramirez E, Brandt M. Mineral Properties and Dietary Value of Raw and Processed Stinging Nettle (Urtica dioica L). Int J Food Sci. 2013;2013:857120. 10.1155/2013/857120. PMC4745470 26904610

[10] Đurović S, Kojić I, Radić D, Smyatskaya YA, Bazarnova JG, Filip S, et al. Chemical Constituents of Stinging Nettle (Urtica dioica L.): A Comprehensive Review on Phenolic and Polyphenolic Compounds and Their Bioactivity. Int J Mol Sci. 2024;25:3430. 10.3390/IJMS25063430. PMC10970493 38542403

[11] Bourgeois C, Leclerc ÉA, Corbin C, Doussot J, Serrano V, Vanier JR, et al. Nettle (Urtica dioica L.) as a source of antioxidant and anti-aging phytochemicals for cosmetic applications. C R Chim. 2016;19:1090–100. 10.1016/J.CRCI.2016.03.019.

[12] Rutkowska N, Drożdżyński P, Ryngajłło M, Marchut-Mikołajczyk O. Plants as the Extended Phenotype of Endophytes—The Actual Source of Bioactive Compounds. Int J Mol Sci. 2023;24:10096. 10.3390/IJMS241210096. PMC10298476 37373241

[13] Afzal I, Shinwari ZK, Sikandar S, Shahzad S. Plant beneficial endophytic bacteria: Mechanisms, diversity, host range and genetic determinants. Microbiol Res. 2019;221:36–49. 10.1016/j.micres.2019.02.001. 30825940

[14] Hardoim PR, van Overbeek LS, van Elsas JD. Properties of bacterial endophytes and their proposed role in plant growth. Trends Microbiol. 2008;16:463–71. 10.1016/J.TIM.2008.07.008. 18789693

[15] Semenzato G, Fani R. Endophytic bacteria: a sustainable strategy for enhancing medicinal plant cultivation and preserving microbial diversity. Front Microbiol. 2024;15:1477465. 10.3389/FMICB.2024.1477465. PMC11609176 39624715

[16] Damam M, Kaloori K, Gaddam B, Kausar R. Plant growth promoting substances (phytohormones) produced by rhizobacterial strains isolated from the rhizosphere of medicinal plants. Int J Pharm Sci Rev Res. 2016;37:130–6.

[17] Eid AM, Fouda A, Abdel-rahman MA, Salem SS, Elsaied A, Oelmüller R, et al. Harnessing Bacterial Endophytes for Promotion of Plant Growth and Biotechnological Applications: An Overview. Plants. 2021;10:935. 10.3390/PLANTS10050935. PMC8151188 34067154

[18] Mercado-Blanco J, Lugtenberg B. Biotechnological Applications of Bacterial Endophytes. Curr Biotechnol. 2014;3:60–75. 10.2174/22115501113026660038.

[19] Kiruba NJM, Thatheyus AJ Fungi, fungal enzymes and their potential application as biostimulants. Microbiome Stimulants Crops. 2021. 10.1016/B978-0-12-822122-8.00024-8.

[20] Timofeeva AM, Galyamova MR, Sedykh SE. Plant Growth-Promoting Soil Bacteria: Nitrogen Fixation, Phosphate Solubilization, Siderophore Production, and Other Biological Activities. Plants. 2023;12:4074. 10.3390/PLANTS12244074. PMC10748132 38140401

[21] Rabbee MF, Ali MS, Islam MN, Rahman MM, Hasan MM, Baek KH. Endophyte mediated biocontrol mechanisms of phytopathogens in agriculture. Res Microbiol. 2024;175:104229. 10.1016/J.RESMIC.2024.104229. 38992820

[22] Lacava PT, Azevedo JL Endophytic bacteria: A biotechnological potential in agrobiology system. Bacteria Agrobiology Crop Productivity. 2013. 10.1007/978-3-642-37241-4_1.

[23] Khalil AMA, Hassan SE-D, Alsharif SM, Eid AM, El-Din Ewais E, Azab E, et al. Isolation and Characterization of Fungal Endophytes Isolated from Medicinal Plant Ephedra pachyclada as Plant Growth-Promoting. Biomolecules. 2021;11:140. 10.3390/BIOM11020140. PMC7911138 33499067

[24] Tian BY, Cao Y, Zhang KQ. Metagenomic insights into communities, functions of endophytes and their associates with infection by root-knot nematode, Meloidogyne incognita, in tomato roots. Sci Rep 2015. 2015;5:1. 10.1038/srep17087. PMC4658523 26603211

[25] Adeleke BS, Muller D, Babalola OO. A metagenomic lens into endosphere microbial communities, promises, and discoveries. Lett Appl Microbiol. 2023;76:1–10. 10.1093/LAMBIO/OVAC030. 36794885

[26] Marchut-Mikołajczyk O, Chlebicz M, Kawecka M, Michalak A, Prucnal F, Nielipinski M, et al. Endophytic bacteria isolated from Urtica dioica L.- preliminary screening for enzyme and polyphenols production. Microb Cell Fact. 2023;22:169. 10.1186/S12934-023-02167-2. PMC10466763 37649058

[27] Marchut-Mikolajczyk O, Drozdzyński P, Pietrzyk D, Antczak T Biosurfactant production and hydrocarbon degradation activity of endophytic bacteria isolated from Chelidonium majus L. Microb Cell Fact. 2018. 10.1186/s12934-018-1017-5. PMC6215600 30390702

[28] Beckers B, Op De Beeck M, Thijs S, Truyens S, Weyens N, Boerjan W, et al. Performance of 16s rDNA primer pairs in the study of rhizosphere and endosphere bacterial microbiomes in metabarcoding studies. Front Microbiol. 2016;7:177245. 10.3389/FMICB.2016.00650. PMC4865482 27242686

[29] Wood DE, Lu J, Langmead B. Improved metagenomic analysis with Kraken 2. Genome Biol. 2019;20:1–13. 10.1186/S13059-019-1891-0. PMC6883579 31779668

[30] Oksanen J, Simpson GL, Blanchet FG, Kindt R, Legendre P, Minchin PR, et al. Community Ecology Package [R package vegan version 2.7-1]. CRAN: Contributed Packages. 2025. 10.32614/CRAN.PACKAGE.VEGAN.

[31] Murtagh F, Legendre P. Ward’s Hierarchical Agglomerative Clustering Method: Which Algorithms Implement Ward’s Criterion? J Classif. 2014;31:274–95. 10.1007/S00357-014-9161-Z.

[32] Lê S, Josse J, Husson F, FactoMineR. An R Package for Multivariate Analysis. J Stat Softw. 2008;25:1–18. 10.18637/JSS.V025.I01.

[33] Kassambara A, Mundt F. Extract and Visualize the Results of Multivariate Data Analyses [R package factoextra version 1.0.7]. CRAN: Contributed Packages. 2020. 10.32614/CRAN.PACKAGE.FACTOEXTRA

[34] Kolde R. Pretty Heatmaps [R package pheatmap version 1.0.13]. CRAN: Contributed Packages. 2025. 10.32614/CRAN.PACKAGE.PHEATMAP

[35] Wickham H. ggplot2. 2nd edition. Springer Cham; 2016. 10.1007/978-3-319-24277-4

[36] Wickham H, Averick M, Bryan J, Chang W, D’ L, Mcgowan A, et al. Welcome to the Tidyverse. J Open Source Softw. 2019;4:1686. 10.21105/JOSS.01686.

[37] Oliveros JC. Venny 2.1.0. An interactive tool for comparing lists with Venn’s diagrams. 2015. https://bioinfogp.cnb.csic.es/tools/venny/. Accessed 13 May 2025.

[38] Antolak H, Oracz J, Otlewska A, Zyzelewicz D, Kregiel D Identification of Carotenoids and Isoprenoid Quinones from Asaia lannensis and Asaia bogorensis. Molecules. 2017; 10.3390/molecules22101608. PMC6151773 28946700

[39] Patkar S, Shinde Y, Chindarkar P, Chakraborty P. Evaluation of antioxidant potential of pigments extracted from Bacillus spp. and Halomonas spp. isolated from mangrove rhizosphere. BioTechnologia. 2021;102:157. 10.5114/BTA.2021.106522. PMC9642923 36606025

[40] Xiao F, Xu T, Lu B, Liu R. Guidelines for antioxidant assays for food components. Food Front. 2020;1:60–9. 10.1002/FFT2.10.

[41] Altschul SF, Gish W, Miller W, Myers EW, Lipman DJ. Basic local alignment search tool. J Mol Biol. 1990;215:403–10. 10.1016/S0022-2836(05)80360-2. 2231712

[42] Tamura K, Stecher G, Kumar S. MEGA11: Molecular Evolutionary Genetics Analysis Version 11. Mol Biol Evol. 2021;38:3022–7. 10.1093/molbev/msab120. PMC8233496 33892491

[43] Saitou N, Nei M. The neighbor-joining method: a new method for reconstructing phylogenetic trees. Mol Biol Evol. 1987;4:406–25. 10.1093/OXFORDJOURNALS.MOLBEV.A040454. 3447015

[44] Passari AK, Mishra VK, Saikia R, Gupta VK, Singh BP. Isolation, abundance and phylogenetic affiliation of endophytic actinomycetes associated with medicinal plants and screening for their in vitro antimicrobial biosynthetic potential. Front Microbiol. 2015;6:129090. 10.3389/FMICB.2015.00273. PMC4388002 25904906

[45] Abdelshafy Mohamad OA, Ma JB, Liu YH, Zhang D, Hua S, Bhute S, et al. Beneficial Endophytic Bacterial Populations Associated With Medicinal Plant Thymus vulgaris Alleviate Salt Stress and Confer Resistance to Fusarium oxysporum. Front Plant Sci. 2020;11:47. 10.3389/FPLS.2020.00047. PMC7033553 32117385

[46] Liu JQ, Chen SM, Zhang CM, Xu MJ, Xing K, Li CG, et al. Abundant and diverse endophytic bacteria associated with medicinal plant Arctium lappa L. and their potential for host plant growth promoting. Antonie van Leeuwenhoek. Int J Gen Mol Microbiol. 2022;115:1405–20. 10.1007/S10482-022-01785-X. 36284037

[47] Frank AC, Guzmán JPS, Shay JE. Transmission of Bacterial Endophytes. Microorganisms. 2017;5:70. 10.3390/MICROORGANISMS5040070. PMC5748579 29125552

[48] Kandel SL, Joubert PM, Doty SL. Bacterial Endophyte Colonization and Distribution within Plants. Microorganisms. 2017;5:77. 10.3390/MICROORGANISMS5040077. PMC5748586 29186821

[49] Chiellini C, Lombardo K, Mocali S, Miceli E, Fani R. Pseudomonas strains isolated from different environmental niches exhibit different antagonistic ability. Ethol Ecol Evol. 2019;31:399–420. 10.1080/03949370.2019.1621391.

[50] Wu H, Cui H, Fu C, Li R, Qi F, Liu Z, et al. Unveiling the crucial role of soil microorganisms in carbon cycling: A review. Sci Total Environ. 2024;909:168627. 10.1016/J.SCITOTENV.2023.168627. 37977383

[51] Lindgren Å, Karami N, Karlsson R, Åhrén C, Welker M, Moore ERB, et al. Development of a rapid MALDI-TOF MS based epidemiological screening method using MRSA as a model organism. Eur J Clin Microbiol Infect Dis. 2017;37:57. 10.1007/S10096-017-3101-X. PMC5748427 28924947

[52] Pfaller MA, Diekema DJ, Gibbs DL, Newell VA, Nagy E, Dobiasova S, et al. Candida krusei, a Multidrug-Resistant Opportunistic Fungal Pathogen: Geographic and Temporal Trends from the ARTEMIS DISK Antifungal Surveillance Program, 2001 to 2005. J Clin Microbiol. 2007;46:515. 10.1128/JCM.01915-07. PMC2238087 18077633

[53] Carrasco G, de Dios Caballero J, Garrido N, Valdezate S, Cantón R, Sáez-Nieto JA. Shortcomings of the commercial MALDI-TOF MS database and use of MLSA as an arbiter in the identification of Nocardia species. Front Microbiol. 2016;7:179559. 10.3389/FMICB.2016.00542. PMC4838697 27148228

[54] Taulé C, Lima A, Beracochea M, Durán R, Battistoni F Modulation of the endophytic strain Kosakonia radicincitans UYSO10 proteome by sugarcane root exudates. Plant Soil. 2024; 10.1007/S11104-024-07112-9.

[55] Xiao D, Zhao F, Lv M, Zhang H, Zhang Y, Huang H, et al. Rapid identification of microorganisms isolated from throat swab specimens of community-acquired pneumonia patients by two MALDI-TOF MS systems. Diagn Microbiol Infect Dis. 2012;73:301–7. 10.1016/J.DIAGMICROBIO.2012.04.004. 22633336

[56] Lohmann C, Sabou M, Moussaoui W, Prévost G, Delarbre JM, Candolfi E, et al. Comparison between the Biflex III-Biotyper and the Axima-SARAMIS Systems for Yeast Identification by Matrix-Assisted Laser Desorption Ionization–Time of Flight Mass Spectrometry. J Clin Microbiol. 2013;51:1231. 10.1128/JCM.03268-12. PMC3666800 23390281

[57] Bielen A, Babić I, Vuk Surjan M, Kazazić S, Šimatović A, Lajtner J, et al. Comparison of MALDI-TOF mass spectrometry and 16S rDNA sequencing for identification of environmental bacteria: a case study of cave mussel-associated culturable microorganisms. Environ Sci Pollut Res. 2024;31:21752–64. 10.1007/S11356-024-32537-1. 38393570

[58] Seuylemezian A, Aronson HS, Tan J, Lin M, Schubert W, Vaishampayan P. Development of a custom MALDI-TOF MS database for species-level identification of bacterial isolates collected from spacecraft and associated surfaces. Front Microbiol. 2018;9:339283. 10.3389/FMICB.2018.00780. PMC5968301 29867782

[59] Pinar-Méndez A, Fernández S, Baquero D, Vilaró C, Galofré B, González S, et al. Rapid and improved identification of drinking water bacteria using the Drinking Water Library, a dedicated MALDI-TOF MS database. Water Res. 2021;203:117543. 10.1016/J.WATRES.2021.117543. 34433109

[60] Toubal S, Bouchenak O, Elhaddad D, Yahiaoui K, Boumaza S, Arab K. MALDI-TOF MS detection of endophytic bacteria associated with great nettle (urtica dioica l.), grown in Algeria. Pol J Microbiol. 2018;67:67–72. 10.5604/01.3001.0011.6145. 30015426

[61] Mamarasulov B, Davranov K, Jahan MS, Jabborova D, Nasif O, Ansari MJ, et al. Characterization, enzymatic and biochemical properties of endophytic bacterial strains of the medicinal plant Ajuga turkestanica (Rgl.) Brig (Lamiaceae). J King Saud Univ Sci. 2022;34:102183. 10.1016/J.JKSUS.2022.102183.

[62] Sulistiyani TR, Lisdiyanti P. Identification of endophytic bacteria from Curcuma zedoaria based on protein profile using MALDI-TOF mass spectrometry. Biotropia (Bogor). 2018;25:112–20. 10.11598/BTB.2018.25.2.795.

[63] Wang X, Wu G, Han S, Yang J, He X, Li H Differentiation and Identification of Endophytic Bacteria from Populus Based on Mass Fingerprints and Gene Sequences. Int J Mol Sci. 2023; 10.3390/IJMS241713449. PMC10487577 37686254

[64] Liu TH, Zhou Y, Tao WC, Liu Y, Zhang XM, Tian SZ. Bacterial diversity in roots, stems, and leaves of Chinese medicinal plant Paris polyphylla var. Yunnanensis. Pol J Microbiol. 2020;69:91–7. 10.33073/PJM-2020-012. PMC7256839 32189484

[65] Alsaedi ZS, Ashy RA, Shami AY, Majeed MA, Alswat AM, Baz L, et al. Metagenomic study of the communities of bacterial endophytes in the desert plant Senna Italica and their role in abiotic stress resistance in the plant. Brazilian J Biology. 2022;82:e267584. 10.1590/1519-6984.267584. 36449831

[66] Hallmann J, Berg G. Spectrum and Population Dynamics of Bacterial Root Endophytes. In: Schulz BJE, Boyle CJC, Sieber TN, editors. Microbial Root Endophytes. 1st edition. Berlin: Springer; 2006. pp. 15–31. 10.1007/3-540-33526-9_2

[67] Hofer U. The majority is uncultured. Nat Rev Microbiol. 2018;16:716–7 10.1038/s41579-018-0097-x 30275521

[68] Moraga D, Latorre K, Muñoz-Torres P, Cárdenas S, Jofré-Quispe A, López-Cepeda J, et al. Diversity of Culturable Bacteria from Endemic Medicinal Plants of the Highlands of the Province of Parinacota. Chile Biology (Basel). 2023;12:920. 10.3390/BIOLOGY12070920/S1. PMC10376134 37508351

[69] Gao L, Ma JB, Huang Y, Muhammad M, Lian HT, Shurigin V, et al. Insight into endophytic microbial diversity in two halophytes and plant beneficial attributes of Bacillus swezeyi. Front Microbiol. 2024;15:1447755. 10.3389/FMICB.2024.1447755. PMC11391308 39268535

[70] Naoufal D, Amine H, Ilham B, Khadija O. Isolation and Biochemical Characterisation of Endophytic Bacillus spp from Urtica dioica and Study of Their Antagonistic Effect against Phytopathogens. Annu Res Rev Biol. 2018;28:1–7. 10.9734/arrb/2018/43642.

[71] Naoufal D, Ilham B, Amine H, Khadija O. Isolation and Characterisation of Endophytic Strain Paenibacillus polymyxa SR19 from Urtica dioica and the Study of Their Effect against Fusarium oxysporum f. sp Tomato. Annu Res Rev Biol. 2018;29:1–8. 10.9734/arrb/2018/44746.

[72] Stamenov D, Đurić S, Hajnal J, Hasanagić D. Plant growth promoting potential of Bacillus, Azotobacter and Streptomyces bacteria from nettle rhizospheric soil. Acta agriculturae Serbica. 2024;29:35–41. 10.5937/aaser2357035s.

[73] Viktorova J, Jandova Z, Madlenakova M, Prouzova P, Bartunek V, Vrchotova B, et al Native phytoremediation potential of Urtica dioica for removal of PCBs and heavy metals can be improved by genetic manipulations using constitutive CaMV 35S promoter. PLoS ONE. 2016; 10.1371/journal.pone.0167927. PMC5145202 27930707

[74] Wang X, He S-W, He Q, Ju Z-C, Ma Y-N, Wang Z, et al Early inoculation of an endophyte alters the assembly of bacterial communities across rice plant growth stages. Microbiol Spectr. 2023; 10.1128/spectrum.04978-22. PMC10580921 37655928

[75] Jechalke S, Schierstaedt J, Becker M, Flemer B, Grosch R, Smalla K, et al. Salmonella establishment in agricultural soil and colonization of crop plants depend on soil type and plant species. Front Microbiol. 2019;10:967. 10.3389/FMICB.2019.00967. PMC6529577 31156568

[76] Michael, Waturangi DE. Antibiofilm activity from endophyte bacteria, Vibrio cholerae strains, and actinomycetes isolates in liquid and solid culture. BMC Microbiol. 2023;23:1–11. 10.1186/S12866-023-02829-6. PMC10053847 36991312

[77] Biswas S, Philip I, Jayaram S, Sarojini S. Endophytic bacteria Klebsiella spp. and Bacillus spp. from Alternanthera philoxeroides in Madiwala Lake exhibit additive plant growth-promoting and biocontrol activities. J Genetic Eng Biotechnol. 2023;21:153. 10.1186/S43141-023-00620-8. PMC10686955 38030944

[78] Martínez-Rodríguez JDC, De La Mora-Amutio M, Plascencia-Correa LA, Audelo-Regalado E, Guardado FR, Hernández-Sánchez E, et al. Cultivable endophytic bacteria from leaf bases of Agave tequilana and their role as plant growth promoters. Brazilian J Microbiol. 2015;45:1333. 10.1590/S1517-83822014000400025. PMC4323307 25763038

[79] Fadiji AE, Ayangbenro AS, Babalola OO. Draft Genome Sequence of Citrobacter freundii AYS58, a Potential Plant Growth-Promoting Endophyte. Microbiol Resour Announc. 2023;12:e00142–23. 10.1128/MRA.00142-23. PMC10190611 37102871

[80] Ahmed A, He P, He Y, Singh BK, Wu Y, Munir S, et al. Biocontrol of plant pathogens in omics era—with special focus on endophytic bacilli. Crit Rev Biotechnol. 2024;44:562–80. 10.1080/07388551.2023.2183379. 37055183

[81] Singh R, Dubey AK. Diversity and Applications of Endophytic Actinobacteria of Plants in Special and Other Ecological Niches. Front Microbiol. 2018;9:1767. 10.3389/FMICB.2018.01767. PMC6092505 30135681

[82] Singh RR, Wesemael WML. Endophytic Paenibacillus polymyxa LMG27872 inhibits Meloidogyne incognita parasitism, promoting tomato growth through a dose-dependent effect. Front Plant Sci. 2022;13:961085. 10.3389/FPLS.2022.961085. PMC9516289 36186028

[83] Verma SK, Chen Q, White JF. Evaluation of colonization and mutualistic endophytic symbiosis of Escherichia coli with tomato and Bermuda grass seedlings. PeerJ. 2022;10:e13879. 10.7717/PEERJ.13879. PMC9375544 35971430

[84] Lopes R, Tsui S, Gonçalves PJRO, de Queiroz MV. A look into a multifunctional toolbox: endophytic Bacillus species provide broad and underexploited benefits for plants. World J Microbiol Biotechnol. 2018;34:1–10. 10.1007/s11274-018-2479-7. 29900507

[85] Grady EN, MacDonald J, Liu L, Richman A, Yuan ZC Current knowledge and perspectives of Paenibacillus: a review. Microb Cell Fact. 2016; 10.1186/S12934-016-0603-7. PMC5134293 27905924

[86] Cho W, Il, Chung MS. Bacillus spores: a review of their properties and inactivation processing technologies. Food Sci Biotechnol. 2020;29:1447. 10.1007/S10068-020-00809-4. PMC7538368 33041624

[87] Marchut-Mikołajczyk O, Drożdżyński P, Polewczyk A, Smułek W, Antczak T. Biosurfactant from endophytic Bacillus pumilus 2A: physicochemical characterization, production and optimization and potential for plant growth promotion. Microb Cell Fact. 2021;20:40. 10.1186/s12934-021-01533-2. PMC7871564 33557838

[88] Nalli Y, Singh S, Gajjar A, Mahizhaveni B, Dusthackeer VNA, Shinde PB Bacillibactin class siderophores produced by the endophyte Bacillus subtilis NPROOT3 as antimycobacterial agents. Lett Appl Microbiol. 2023; 10.1093/LAMBIO/OVAC026. 36794884

[89] Yin QJ, Ying TT, Zhou ZY, Hu GA, Yang CL, Hua Y, et al. Species-specificity of the secondary biosynthetic potential in Bacillus. Front Microbiol. 2023;14:1271418. 10.3389/FMICB.2023.1271418. PMC10626522 37937215

[90] Shida O, Takagi H, Kadowaki K, Nakamura LK, Komagata K. Transfer of Bacillus alginolyticus, Bacillus chondroitinus, Bacillus curdlanolyticus, Bacillus glucanolyticus, Bacillus kobensis, and Bacillus thiaminolyticus to the genus Paenibacillus and emended description of the genus Paenibacillus. Int J Syst Bacteriol. 1997;47:289–98. 10.1099/00207713-47-2-289. 9103612

[91] Taheri E, Tarighi S, Taheri P. Characterization of root endophytic Paenibacillus polymyxa isolates with biocontrol activity against Xanthomonas translucens and Fusarium graminearum. Biol Control. 2022;174:105031. 10.1016/J.BIOCONTROL.2022.105031.

[92] Poehlein A, Hollensteiner J, Granzow S, Wemheuer B, Vidal S, Wemheuer F First Insights into the Draft Genome Sequence of the Endophyte Paenibacillus amylolyticus Strain GM1FR, Isolated from Festuca rubra L. Genome Announc. 2018; 10.1128/GENOMEA.01516-17. PMC5786679 29371353

[93] Radhakrishnan R, Ajithkumar P, Arun M, Sathasivam R, Sandhya S, Choi J, et al. An endophyte Paenibacillus dendritiformis strain APL3 promotes Amaranthus polygonoides L. sprout growth and their extract inhibits food-borne pathogens. Plant Sci Today. 2021;8:941–7. 10.14719/PST.2021.8.4.1259.

[94] Carro L, Flores-Félix JD, Ramírez-Bahena MH, García-Fraile P, Martínez-Hidalgo P, Igual JM, et al. Paenibacillus lupini sp. nov., Isolated from nodules of Lupinus albus. Int J Syst Evol Microbiol. 2014;64:3028–33. 10.1099/IJS.0.060830-0. 24928428

[95] Elo S, Suominen I, Kämpfer P, Juhanoja J, Salkinoja-Salonen M, Haahtela K. Paenibacillus borealis sp. nov., a nitrogen-fixing species isolated from spruce forest humus in Finland. Int J Syst Evol Microbiol. 2001;51:535–45. 10.1099/00207713-51-2-535. 11321100

[96] Carro L, Flores-Félix JD, Cerda-Castillo E, Ramírez-Bahena MH, Igual JM, Tejedor C, et al. Paenibacillus endophyticus sp. nov., isolated from nodules of Cicer arietinum. Int J Syst Evol Microbiol. 2013;63:4433–8. 10.1099/IJS.0.050310-0. 23852155

[97] Bhattacharya E, Hazra A, Dutta M, Bose R, Dutta A, Dandapat M, et al. Novel report of Acinetobacter johnsonii as an indole-producing seed endophyte in Tamarindus indica L. Arch Microbiol. 2024;206:1–10. 10.1007/S00203-024-03865-0. 38460008

[98] Yuan QS, Deng T, Gao Y, Jiang W, Ou X, Wang Y, et al. Genome Resource for Acinetobacter schindleri H4-3-C1: An Endophyte of Pseudostellaria heterophylla with Degradation Activity to Toxins Produced by Fungal Pathogens. Mol Plant Microbe Interact. 2022;35:1124–6. 10.1094/MPMI-04-22-0088-A. 36508486

[99] Amer MA, Wasfi R, Hamed SM. Biosurfactant from Nile Papyrus endophyte with potential antibiofilm activity against global clones of Acinetobacter baumannii. Front Cell Infect Microbiol. 2023;13:1210195. 10.3389/FCIMB.2023.1210195. PMC10373939 37520441

[100] da Silva Bandeira ON, da Silva Bandeira R, de Souza CRB. Systematic review and meta-analysis of the potential effects of endophytic bacteria Klebsiella on plant growth promotion and biocontrol of pathogens. World J Microbiol Biotechnol. 2025;41:1–16. 10.1007/S11274-025-04300-2. 40021542

[101] Amenta ML, Varriale S, Arbucci S, Fania F, De Vita P, Summerer S, et al. The endophyte Klebsiella pasteurii BDA134-6 isolated from African rice colonizes durum wheat plants helping them cope with water shortage stress. Sci Rep. 2025;15:1–17. 10.1038/S41598-025-13827-1. PMC12321996 40760159

[102] Ou T, Gao H, Jiang K, Yu J, Zhao R, Liu X, et al. Endophytic Klebsiella aerogenes HGG15 stimulates mulberry growth in hydro-fluctuation belt and the potential mechanisms as revealed by microbiome and metabolomics. Front Microbiol. 2022;13:978550. 10.3389/FMICB.2022.978550. PMC9417544 36033884

[103] Tharek M, Khairuddin D, Najimudin N, Ghazali AH. Plant Growth Promoting Potentials of Beneficial Endophytic Escherichia coli USML2 in Association with Rice Seedlings. Trop Life Sci Res. 2021;32:119. 10.21315/TLSR2021.32.1.8. PMC8054666 33936555

[104] Švec P, Vandamme P, Bryndová H, Holochová P, Kosina M, Mašlaňová I, et al. Enterococcus plantarum sp. nov., isolated from plants. Int J Syst Evol Microbiol. 2012;62:1499–505. 10.1099/IJS.0.033357-0. 21856982

[105] Kljujev I, Raicevic V, Vujovic B, Rothballer M, Schmid M. Salmonella as an endophytic colonizer of plants - A risk for health safety vegetable production. Microb Pathog. 2018;115:199–207. 10.1016/j.micpath.2017.12.020. 29248516

[106] An SQ, Potnis N, Dow M, Vorhölter FJ, He YQ, Becker A, et al. Mechanistic insights into host adaptation, virulence and epidemiology of the phytopathogen Xanthomonas. FEMS Microbiol Rev. 2019;44:1. 10.1093/FEMSRE/FUZ024. PMC8042644 31578554

[107] Timilsina S, Potnis N, Newberry EA, Liyanapathiranage P, Iruegas-Bocardo F, White FF, et al. Xanthomonas diversity, virulence and plant–pathogen interactions. Nat Rev Microbiol. 2020;18:415–27. 10.1038/S41579-020-0361-8. 32346148

[108] Becker A, Vorhölter F-J. Xanthan biosynthesis by Xanthomonas bacteria: an overview of the current biochemical and genomic data. In: Rehm BHA, editor. Microbial production of biopolymers and polymer precursors: applications and perspectives. Caister Academic; 2009. pp. 1–11.

[109] Rana R, Nayak PK, Madhavan VN, Sonti RV, Patel HK, Patil PB Comparative genomics-based insights into Xanthomonas indica, a non-pathogenic species of healthy rice microbiome with bioprotection function. Appl Environ Microbiol. 2024. 10.1128/AEM.00848-24. PMC11409687 39158313

[110] Furtado BU, Gołebiewski M, Skorupa M, Hulisz P, Hrynkiewicz K. Bacterial and Fungal Endophytic Microbiomes of Salicornia europaea. Appl Environ Microbiol. 2019;85:e00305–19. 10.1128/AEM.00305-19. PMC6581177 31003988

[111] Selvankumar T, Radhika R, Mythili R, Arunprakash S, Srinivasan P, Govarthanan M, et al Isolation, identification and characterization of arsenic transforming exogenous endophytic Citrobacter sp. RPT from roots of Pteris vittata. 3 Biotech. 2017. 10.1007/s13205-017-0901-8. PMC5529295 28794920

[112] Li FN, Tuo L, Pan Z, Guo M, Lee SMY, Chen L, et al. Aureimonas endophytica sp. nov., a novel endophytic bacterium isolated from Aegiceras corniculatum. Int J Syst Evol Microbiol. 2017;67:2934–40. 10.1099/ijsem.0.002046. 28820121

[113] Liaqat F, Eltem R Identification and characterization of endophytic bacteria isolated from in vitro cultures of peach and pear rootstocks. 3 Biotech. 2016; 10.1007/s13205-016-0442-6. PMC4909027 28330195

[114] Manjunatha BS, Nivetha N, Krishna GK, Elangovan A, Pushkar S, Chandrashekar N, et al Plant growth-promoting rhizobacteria Shewanella putrefaciens and Cronobacter dublinensis enhance drought tolerance of pearl millet by modulating hormones and stress-responsive genes. Physiol Plant. 2022; 10.1111/ppl.13676. 35316540

[115] Barreto JV, de Casanova O, Junior LM, Reis-Mansur AN, Vermelho MCPP. Microbial Pigments: Major Groups and Industrial Applications. Microorganisms. 2023;11:2920. 10.3390/MICROORGANISMS11122920 PMC10745774 38138065

[116] Narsing Rao MP, Xiao M, Li WJ. Fungal and bacterial pigments: Secondary metabolites with wide applications. Front Microbiol. 2017;8:1113. 10.3389/fmicb.2017.01113. PMC5479939 28690593

[117] Muhammad M, Basit A, Majeed M, Shah AA, Ullah I, Mohamed HI, et al. Bacterial pigments and their applications. Bacterial Secondary Metabolites: Synthesis and Applications in Agroecosystem. Elsevier; 2023. pp. 277–98. 10.1016/B978-0-323-95251-4.00019-3.

[118] Mummaleti G, Udo T, Mohan A, Kong F. Synthesis, characterization and application of microbial pigments in foods as natural colors. Crit Rev Food Sci Nutr. 2025;65:5602–31. 10.1080/10408398.2024.2417802. 39466660

[119] Morna A. Chlorophyll and carotenoid content in lettuce (Lactuca Sativa L.) and nettle leaves (Urtica Dioica L.). Analele Universităţii din Oradea, Fascicula: Ecotoxicologie, Zootehnie şi Tehnologii de Industrie Alimntară. 2015;14:243–8.

[120] Hagaggi NSA, Mohamed AAA. Plant–bacterial endophyte secondary metabolite matching: a case study. Arch Microbiol. 2020;202:2679–87. 10.1007/S00203-020-01989-7. 32719949

[121] Khanam B, Chandra R. Isolation and identification of endophytic bacteria producing bright red pigment from the dye yielding plant Beta vulgaris l. Int J Pharm Pharm Sci. 2015;7:220–4.

[122] Meyer JM, Abdallah MA. The fluorescent pigment of Pseudomonas fluorescens: Biosynthesis, purification and physicochemical properties. J Gen Microbiol. 1978;107:319–28. 10.1099/00221287-107-2-319.

[123] Choi O, Kim J, Kim JG, Jeong Y, Jae SM, Chang SP, et al. Pyrroloquinoline quinone is a plant growth promotion factor produced by Pseudomonas fluorescens B16. Plant Physiol. 2008;146:657–68. 10.1104/pp.107.112748. PMC2245851 18055583

[124] Fang T, Lo SC, Yu YN, Sou NL, Hung SHW, Peng JH, et al. Development of an Engineered Bacterial Endophyte: Promoting Plant Growth Through Pyrroloquinoline Quinone (PQQ) Synthesis. Microorganisms. 2025;13:293. 10.3390/MICROORGANISMS13020293. PMC11858353 40005660

[125] Hagaggi NSA, Abdul-Raouf UM Production of bioactive β-carotene by the endophytic bacterium Citricoccus parietis AUCs with multiple in vitro biological potentials. Microb Cell Fact. 2023; 10.1186/s12934-023-02108-z. PMC10155329 37138322

[126] Kandasamy GD, Kathirvel P Production, characterization and in vitro biological activities of crude pigment from endophytic Micrococcus luteus associated with Avicennia marina. Arch Microbiol. 2024; 10.1007/s00203-023-03751-1. 38108901

[127] Gopal JV, Subashini E, Kannabiran K. Extraction of quinone derivative from Streptomyces sp. VITVSK1 isolated from Cheyyur saltpan, Tamilnadu, India. J Korean Soc Appl Biol Chem. 2013;56:361–7. 10.1007/S13765-013-3052-6.

[128] Ruivo M, Cartaxana P, Cardoso MI, Tenreiro A, Tenreiro R, Jesus B. Extraction and quantification of pigments in aerobic anoxygenic phototrophic bacteria. Limnol Oceanogr Methods. 2014;12:338–50. 10.4319/LOM.2014

[129] Leyva E, Schmidtke Sobeck SJ, Loredo-Carrillo SE, Magaldi-Lara DA. Spectral and structural characterization of 2-(fluorophenylamino)- and 2-(nitrophenylamino)-1,4-naphthoquinone derivatives. J Mol Struct. 2014;1068:1–7. 10.1016/J.MOLSTRUC.2014.03.044.

[130] Hadibarata T, Teh ZC, Rubiyatno, Zubir MMFA, Khudhair AB, Yusoff ARM, et al. Identification of naphthalene metabolism by white rot fungus Pleurotus eryngii. Bioprocess Biosyst Eng. 2013;36:1455–61. 10.1007/S00449-013-0884-8. 23334282

[131] Samiee F, Pedron FN, Estrin DA, Trevani L. Experimental and Theoretical Study of the High-Temperature UV-Visible Spectra of Aqueous Hydroquinone and 1,4-Benzoquinone. J Phys Chem B. 2016;120:10547–52. 10.1021/ACS.JPCB.6B07893. 27627463

[132] Pauli FP, Freitas CS, Pereira PR, Magalhães A, de Carvalho da Silva F, Paschoalin VMF, et al. Exploring the Antimicrobial and Antitumoral Activities of Naphthoquinone-Grafted Chitosans. Polym (Basel). 2023;15:1430. 10.3390/POLYM15061430. PMC10053705 36987212

[133] Li F, Xue F, Yu XH. GC–MS, FTIR and Raman Analysis of Antioxidant Components of Red Pigments from Stemphylium lycopersici. Curr Microbiol. 2017;74:532–9. 10.1007/S00284-017-1220-3. 28255784

[134] Soliman IA, Hasanien YA, Zaki AG, Shawky HA, Nassrallah AA. Irradiation impact on biological activities of Anthraquinone pigment produced from Talaromyces purpureogenus and its evaluation, characterization and application in beef burger as natural preservative. BMC Microbiol. 2022;22:1–18. 10.1186/S12866-022-02734-4. PMC9801527 36581795

[135] Venil CK, Zakaria ZA, Ahmad WA. Bacterial pigments and their applications. Process Biochem. 2013;48:1065–79. 10.1016/j.procbio.2013.06.006.

[136] Chandra P, Sharma RK, Arora DS. Antioxidant compounds from microbial sources: A review. Food Res Int. 2020;129:108849. 10.1016/J.FOODRES.2019.108849. 32036890

[137] Mendonça JdaS, Guimarães R, de CA, Zorgetto-Pinheiro VA, Fernandes CD, Pietro, Marcelino G, Bogo D, et al. Natural Antioxidant Evaluation: A Review of Detection Methods. Molecules. 2022;27:3563. 10.3390/MOLECULES27113563. PMC9182375 35684500

