# Supplementary material for: Integrating culture-dependent and culture-independent approaches to explore the diversity of endophytic bacteria associated with Urtica dioica L. in Poland with screening of pigment-producing isolates
Source: Microb Cell Fact. 2026 Aug 4;25:229. doi: 10.1186/s12934-026-03077-9 (PMC13729438; doi:10.1186/s12934-026-03077-9)
Supplement: Supplementary file 1 [file 12934_2026_3077_MOESM1_ESM.docx]

**Supplementary data**

**Table S1**. Abundance table for the phylum rank

| **Phylum** | **CP_L** | **NP_R** | **NP_S** | **NP_L** | **SP_S** | **WP_R** | **WP_L** | **EP_R** | **EP_S** | **EP_L** |
| --- | --- | --- | --- | --- | --- | --- | --- | --- | --- | --- |
|  | % | | | | | | | | | |
| Unknown | 71.68 | 70.85 | 70.90 | 93.11 | 73.23 | 59.61 | 86.56 | 74.58 | 64.66 | 83.94 |
| ***Actinomycetota*** | **0.19** | **6.66** | **8.66** | **0.07** | **0.37** | **3.75** | **0.04** | **3.44** | **0.15** | **0.27** |
| ***Bacillota*** | **5.71** | **11.81** | **7.53** | **1.45** | **9.14** | **5.54** | **8.47** | **8.42** | **10.95** | **6.62** |
| *Bacteroidota* | 0.21 | 0.59 | 0.27 | 0.04 | 0.05 | 0.18 | 0.00 | 1.90 | 0.15 | 0.11 |
| *Mycoplasmatota* | 0.00 | 0.03 | 0.01 | 0.00 | 0.00 | 0.00 | 0.00 | 0.04 | 0.00 | 0.00 |
| *Planctomycetota* | 0.03 | 0.14 | 0.03 | 0.00 | 0.01 | 0.11 | 0.03 | 0.05 | 0.12 | 0.00 |
| ***Pseudomonadota*** | **22.09** | **9.54** | **12.46** | **5.31** | **17.10** | **30.69** | **4.78** | **11.31** | **23.90** | **8.89** |
| *Verrucomicrobiota* | 0.00 | 0.04 | 0.01 | 0.00 | 0.01 | 0.02 | 0.00 | 0.14 | 0.01 | 0.00 |
| Other | 0.10 | 0.34 | 0.14 | 0.02 | 0.10 | 0.11 | 0.12 | 0.11 | 0.05 | 0.16 |

* R – root; S – stem; L – leaf

**Table S2**. Abundance table for the genus rank

| **Genus** | **CP_L** | **NP_R** | **NP_S** | **NP_L** | **SP_S** | **WP_R** | **WP_L** | **EP_R** | **EP_S** | **EP_L** |
| --- | --- | --- | --- | --- | --- | --- | --- | --- | --- | --- |
|  | % | | | | | | | | | |
| Unknown | 71.68 | 70.85 | 70.90 | 93.11 | 73.23 | 59.61 | 86.56 | 74.58 | 64.66 | 83.94 |
| ***Acinetobacter*** | 0.29 | 0.13 | 0.09 | 0.01 | 0.54 | 0.93 | 0.12 | 0.02 | 0.27 | 0.02 |
| *Arthrobacter* | 0.00 | 0.40 | 0.39 | 0.00 | 0.00 | 0.08 | 0.00 | 0.07 | 0.03 | 0.00 |
| *Aurantimonas* | 0.31 | 0.00 | 0.04 | 0.00 | 0.02 | 0.00 | 0.00 | 0.01 | 0.00 | 0.00 |
| *Aureimonas* | 1.12 | 0.00 | 0.32 | 0.00 | 1.01 | 2.66 | 0.10 | 0.04 | 0.00 | 0.00 |
| ***Bacillus*** | 4.81 | 5.15 | 3.54 | 0.25 | 2.35 | 4.63 | 0.31 | 3.52 | 10.50 | 0.32 |
| *Brevundimonas* | 0.06 | 0.00 | 0.05 | 0.10 | 0.02 | 0.20 | 0.03 | 0.01 | 0.00 | 0.05 |
| *Brucella* | 0.41 | 1.29 | 1.92 | 0.00 | 4.28 | 2.54 | 0.00 | 0.96 | 0.08 | 0.32 |
| *Burkholderia* | 0.40 | 0.02 | 0.40 | 0.00 | 0.01 | 0.71 | 0.00 | 0.01 | 0.83 | 0.00 |
| *Cellulomonas* | 0.00 | 0.20 | 0.04 | 0.02 | 0.00 | 0.00 | 0.00 | 0.07 | 0.00 | 0.00 |
| *Chryseobacterium* | 0.10 | 0.13 | 0.04 | 0.00 | 0.00 | 0.09 | 0.00 | 0.02 | 0.00 | 0.00 |
| ***Citrobacter*** | 0.41 | 0.16 | 0.12 | 0.01 | 0.22 | 0.16 | 0.25 | 0.22 | 0.20 | 0.18 |
| *Clostridioides* | 0.00 | 2.51 | 0.04 | 0.06 | 0.00 | 0.08 | 0.00 | 0.34 | 0.00 | 1.51 |
| *Clostridium* | 0.32 | 0.21 | 0.26 | 0.00 | 0.01 | 0.08 | 2.17 | 1.05 | 0.05 | 0.02 |
| *Corynebacterium* | 0.04 | 0.08 | 0.23 | 0.00 | 0.00 | 0.04 | 0.00 | 0.04 | 0.00 | 0.00 |
| *Delftia* | 0.19 | 0.25 | 0.27 | 0.00 | 0.04 | 0.12 | 0.00 | 0.31 | 0.00 | 0.07 |
| ***Enterococcus*** | 0.32 | 0.44 | 0.60 | 0.68 | 6.27 | 0.49 | 5.41 | 2.09 | 0.31 | 4.02 |
| ***Escherichia*** | 5.03 | 2.05 | 2.78 | 0.76 | 0.58 | 2.30 | 2.91 | 4.91 | 3.67 | 5.46 |
| ***Klebsiella*** | 1.73 | 0.17 | 0.14 | 0.01 | 0.21 | 0.32 | 0.12 | 0.18 | 0.26 | 0.23 |
| *Liberibacter* | 0.00 | 0.07 | 0.01 | 0.01 | 7.06 | 11.12 | 0.01 | 0.01 | 0.03 | 0.00 |
| *Massilia* | 0.00 | 0.06 | 0.20 | 0.00 | 0.02 | 0.04 | 0.09 | 0.13 | 0.94 | 0.16 |
| *Microbacterium* | 0.00 | 0.50 | 1.00 | 0.00 | 0.13 | 0.04 | 0.01 | 0.02 | 0.00 | 0.00 |
| *Mycobacterium* | 0.01 | 0.10 | 0.05 | 0.00 | 0.00 | 2.18 | 0.00 | 0.02 | 0.00 | 0.00 |
| ***Paenibacillus*** | 0.06 | 0.47 | 0.06 | 0.01 | 0.10 | 0.07 | 0.04 | 0.11 | 0.03 | 0.05 |
| *Pantoea* | 0.45 | 0.02 | 0.01 | 0.01 | 0.00 | 0.00 | 0.00 | 0.01 | 0.03 | 0.00 |
| *Phycisphaera* | 0.01 | 0.01 | 0.01 | 0.00 | 0.00 | 0.06 | 0.03 | 0.01 | 0.12 | 0.00 |
| *Pseudomonas* | 0.70 | 1.42 | 1.21 | 0.00 | 0.72 | 4.25 | 0.09 | 0.40 | 2.39 | 0.53 |
| *Rhizobium* | 0.06 | 0.35 | 0.12 | 0.01 | 0.57 | 0.90 | 0.01 | 0.17 | 0.00 | 0.02 |
| *Rhodococcus* | 0.04 | 0.50 | 0.52 | 0.00 | 0.02 | 0.15 | 0.00 | 0.17 | 0.00 | 0.00 |
| *Roseicitreum* | 0.00 | 0.15 | 0.09 | 0.01 | 0.06 | 0.05 | 0.07 | 0.10 | 0.14 | 0.11 |
| ***Salmonella*** | 5.86 | 0.03 | 0.35 | 0.27 | 0.06 | 0.47 | 0.06 | 0.61 | 0.57 | 0.18 |
| *Serratia* | 0.26 | 0.02 | 0.02 | 0.01 | 0.01 | 0.04 | 0.01 | 0.02 | 0.01 | 0.00 |
| *Shewanella* | 0.01 | 0.02 | 0.03 | 0.00 | 0.01 | 0.05 | 0.01 | 0.02 | 0.11 | 0.00 |
| *Shigella* | 0.21 | 0.31 | 0.19 | 0.04 | 0.00 | 0.14 | 0.00 | 0.37 | 0.15 | 0.00 |
| *Sodalis* | 0.13 | 0.02 | 0.01 | 0.01 | 0.01 | 0.04 | 0.04 | 0.03 | 0.03 | 0.00 |
| *Sphingomonas* | 0.22 | 0.13 | 1.13 | 0.00 | 0.20 | 0.19 | 0.00 | 0.02 | 0.00 | 0.00 |
| *Staphylococcus* | 0.01 | 0.18 | 0.05 | 0.11 | 0.09 | 0.03 | 0.00 | 0.13 | 0.00 | 0.00 |
| *Stenotrophomonas* | 0.04 | 0.08 | 0.00 | 0.00 | 0.00 | 0.08 | 0.00 | 0.07 | 0.03 | 0.02 |
| *Streptococcus* | 0.00 | 1.84 | 2.72 | 0.30 | 0.00 | 0.00 | 0.00 | 0.00 | 0.00 | 0.18 |
| *Streptomyces* | 0.06 | 0.75 | 0.67 | 0.00 | 0.03 | 0.53 | 0.00 | 0.99 | 0.00 | 0.00 |
| ***Vibrio*** | 0.16 | 0.03 | 0.06 | 0.01 | 0.03 | 0.14 | 0.03 | 0.04 | 0.04 | 0.02 |
| ***Xanthomonas*** | 1.13 | 0.61 | 0.51 | 4.04 | 0.41 | 0.30 | 0.67 | 0.30 | 0.22 | 0.85 |
| Other | 3.36 | 8.29 | 8.81 | 0.15 | 1.68 | 4.09 | 0.85 | 7.80 | 14.30 | 1.74 |

**Figure S1.** Diversity of endophytic bacteria found in samples NP (**A, C, E**) and EP (**B, D, F**) divided into tissues: root (**A**, **B**), stem (**C, D**), leaf (**E**, **F**).
